# Two forms of short-interval intracortical inhibition in human motor cortex

**DOI:** 10.1016/j.brs.2021.08.022

**Published:** 2021

**Authors:** Po-Yu Fong, Danny Spampinato, Lorenzo Rocchi, Ricci Hannah, Yinghui Teng, Alessandro Di Santo, Mohamed Shoura, Kailash Bhatia, John C. Rothwell

**Affiliations:** a Department of Clinical and Movement Neurosciences, UCL Queen Square Institute of Neurology, University College London, London, UK; b Division of Movement Disorders, Department of Neurology and Neuroscience Research Center, Chang Gung Memorial Hospital at Linkou, Taoyuan City, Taiwan; c Medical School, College of Medicine, Chang Gung University, Taoyuan, Taiwan; d Non-invasive Brain Stimulation Unit, IRCCS Santa Lucia Foundation, Via Ardeatina 306/354, 00142, Rome, Italy; e Department of Medical Sciences and Public Health, University of Cagliari, Cagliari, Italy; f Department of Psychology, University of California San Diego, 9500 Gilman Drive, La Jolla, CA, 92093, USA; g Division of Biosciences, University College London, London, UK; h Neurology, Neurophysiology and Neurobiology Unit, Department of Medicine, Università Campus Bio-Medico di Roma, Rome, Italy; i Department of Neurology, Heliopolis and Al Azhar University Hospitals, Cairo, Egypt

**Keywords:** SICI, TMS, Coil orientation, Triple pulse stimulation, Movement preparation, Inhibitory circuit

## Abstract

**Background:**

Pulses of transcranial magnetic stimulation (TMS) with a predominantly anterior-posterior (***AP***) or posterior-anterior (PA) current direction over the primary motor cortex appear to activate distinct excitatory inputs to corticospinal neurons. In contrast, very few reports have examined whether the inhibitory neurons responsible for short-interval intracortical inhibition (SICI) are sensitive to TMS current direction.

**Objectives:**

To investigate whether SICI evaluated with ***AP*** and PA conditioning stimuli (CS_PA_ and CS_***AP***_) activate different inhibitory pathways. SICI was always assessed using a PA-oriented test stimulus (TS_PA_).

**Methods:**

Using two superimposed TMS coils, CS_PA_ and CS_***AP***_ were applied at interstimulus intervals (ISI) of 1–5 ms before a TS_PA_, and at a range of different intensities. Using a triple stimulation design, we then tested whether SICI at ISI of 3 ms using opposite directions of CS (SICI_CSPA3_ and SICI_***CSAP3***_) interacted differently with three other forms of inhibition, including SICI at ISI of 2 ms (SICI_CSPA2_), cerebellum-motor cortex inhibition (CBI 5 ms) and short-latency afferent inhibition (SAI 22 ms). Finally, we compared the effect of tonic and phasic voluntary contraction on SICI_CSPA3_ and SICI_***CSAP3***_.

**Results:**

CS_***AP***_ produced little SICI at ISIs = 1 and 2 ms. However, at ISI = 3 ms, both CS_***AP***_ and CS_PA_ were equally effective at the same percent of maximum stimulator output. Despite this apparent similarity, combining SICI_CSPA3_ or SICI_***CSAP3***_ with other forms of inhibition led to quite different results: SICI_CSPA3_ interacted in complex ways with CBI, SAI and SICI_CSPA2_, whereas the effect of SICI_***CSAP3***_ appeared to be quite independent of them. Although SICI_CSPA_ and SICI_***CSAP***_ were both reduced by the same amount during voluntary tonic contraction compared with rest, in a simple reaction time task SICI_***CSAP***_ was disinhibited much earlier following the imperative signal than SICI_CSPA_.

**Conclusions:**

SICI_CSPA_ appears to activate a different inhibitory pathway to that activated by SICI_***CSAP***_. The difference is behaviourally relevant since the pathways are controlled differently during volitional contraction. The results may explain some previous pathological data and open the possibility of testing whether these pathways are differentially recruited in a range of tasks.

## Highlights

• Opposite directions of conditioning stimulus (CS) used to suppress MEPs evoked by a conventional test stimulus.
• Different directions of CS have different time courses of short-interval intracortical inhibition (SICI).
• They also interact differently with short-latency afferent inhibition and with cerebellar inhibition.
• They are differently affected in a reaction time task.
• We suggest there are two forms of SICI in motor cortex.

## 1 Introduction

All movements involve critical interactions between inhibitory and excitatory interneurons within the primary motor cortex (M1). Insights to these interneuronal circuits can be achieved in humans with transcranial magnetic stimulation (TMS) by assessing how different inputs to the cortex influence the excitability of the corticospinal tract via their effects on motor evoked potentials (MEP). Ever since its introduction, it has been known that TMS of the M1 hand area can activate two separate inputs to corticospinal output cells by changing the direction of current from posterior-to-anterior (PA) to anterior-to-posterior (***AP***). The latter evokes MEPs with longer latency and higher threshold [1,2], and the neural elements that it activates have a different strength-duration time constant than those responding to PA stimulation [3]. The source of these two sets of inputs is unknown, but it has been speculated that ***AP*** currents activate inputs from more anterior (premotor) locations than PA currents [[4], [5], [6], [7]].

Double-pulse TMS, in which a subthreshold conditioning stimulus (CS) is followed by a suprathreshold test stimulus (TS) at interstimulus intervals (ISI) of 1–5 ms, can be used to examine a GABAa-ergic cortico-cortical inhibitory process termed short-interval intracortical inhibition (SICI) [8,9]. SICI is implicated, for example, in movement preparation [10,11] and is reduced in a variety of movement disorders, such as dystonia and cortical myoclonus [12,13]. Of note, it is particularly important to use the appropriate CS intensity and ISI, as well as the appropriate intensity of the TS to assess SICI; adjusting these parameters results in different effects that may reflect distinct underlying mechanisms [14,15]. For example, SICI evaluated at an interstimulus interval of 1 ms appears to have quite a different mechanism than at 3 ms [16]; higher intensities of CS can contaminate SICI with a separate phenomenon, short-interval intracortical facilitation [17,18].

Very few papers have investigated whether SICI is sensitive to the orientation of the CS. Conducting these experiments requires either two separate, but overlapping, TMS coils that can be oriented at different angles to the central sulcus, or a special device that allows two stimuli of opposite directions to be delivered through the same coil. Ziemann et al. (1996) [18] used a two-coil approach and found that rotating the CS by 90° to induce latero-medial (CS_LM_-TS_PA_) currents in the brain produced the same amount of SICI as conventional CS in PA direction (CS_PA_-TS_PA_, SICI_PA_). The result is compatible with the idea that, unlike the excitatory elements responsible for the MEP, inhibitory elements have no preferred orientation and therefore can be activated by any direction of CS. Hanajima et al. (1998) [14] delivered oppositely directed CS and TS through the same coil and came to a similar conclusion, although they tested participants during slight muscle contraction rather than at rest.

However, one result suggested that opposite CS directions may recruit two separate mechanisms. Hanajima et al. (2008) [12] found that, although SICI_PA_ was reduced or absent in patients with dystonia, it was normal in the same patients if a SICI_***AP***_ (CS_***AP***_-TS_***AP***_) was used. Thus, SICI_***AP***_ seemed to be pathologically different from SICI_PA_. Unfortunately, Hanajima (2008) [12] did not use a special current reversal device to deliver different directions of CS and TS in this particular set of experiments. Thus, it was never clear whether the dissociation in dystonia was a result of changing the direction of the CS or the TS.

The aim of the present set of experiments was to revisit the orientation sensitivity of SICI using a two-coil method with a constant PA test stimulus (TS_PA_) and ISIs from 1 to 5 ms. The results showed that the time course of SICI differed for CS_***AP***_ and CS_PA_ conditioning stimuli, suggesting that there might be two types of SICI (SICI_CSPA_ (i.e. CS_PA_-TS_PA_) and SICI_***CSAP***_ (i.e. CS_***AP***_-TS_PA_)). To test this further, we made use of the fact that many previous papers have described how SICI interacts with other forms of inhibition such as SICI_CSPA_ with different ISI [19], cerebellar-motor cortex inhibition (CBI) [20], and short-latency afferent inhibition (SAI) [[21], [22], [23]]). Our hypothesis was that if SICI_CSPA_ and SICI_***CSAP***_ had different mechanisms, then they would interact differently with SICI_CSPA_, CBI and SAI. Finally, to test the functional relevance of these two forms of SICI, behavioural experiments provided further evidence that SICI_CSPA_ and SICI_***CSAP***_ have distinct neurophysiological features.

## 2 Materials and methods

### 2.1 Participants

A total of 36 healthy participants (18 males and 18 females; mean age: 25.5 ± 4.5 years) without any neurological or other disease were recruited and consented to participate in the study. All experiments were approved by the University College London Ethics Committee, and all experimental designs followed the international safety guidelines for non-invasive brain stimulation [24]. The numbers of participants in each experiment and the overlap between them are tabulated in supplementary material 4 and 5.

### 2.2 Surface electromyography

Surface electromyography (EMG) signals were recorded using a belly-tendon montage from surface electrodes (WhiteSensor 40713, AmbuR, Denmark) over the right first dorsal interosseous (FDI) muscle. Data were amplified with a gain of 1000, bandpass filtered (5 Hz–3000 Hz) by a Digitimer D360 amplifier (Digitimer Ltd, Welwyn Garden City, Hers, UK), and digitised at 5000 Hz by a Power 1401 data acquisition interface (Cambridge Electronic Design Ltd., Cambridge, UK). All recorded MEPs were stored in the same computer for offline analysis with Signal software version 7.01 (Cambridge Electronic Design Ltd., Cambridge, UK).

### 2.3 Transcranial magnetic stimulation (TMS)

TMS pulses were delivered by Magstim 200 Monophasic stimulators (Magstim Co., UK). Three different coils were used throughout the experiments: (1) a figure-of-eight coil with an outer diameter of 80 mm (D50, Magstim Co., UK), (2) an oval coil with a long outer diameter of 130 mm and a short outer diameter of 100 mm (Magstim Co., UK) and (3) a 110 mm double cone coil (Magstim Co., UK) used solely for cerebellar stimulation. Note that the oval coil was used in preference to a second figure-of-eight coil because it has a thinner profile, reducing the scalp-coil distance of the overlying figure-of-eight coil.

### 2.4 Motor cortex stimulation

**Fig. 1:**
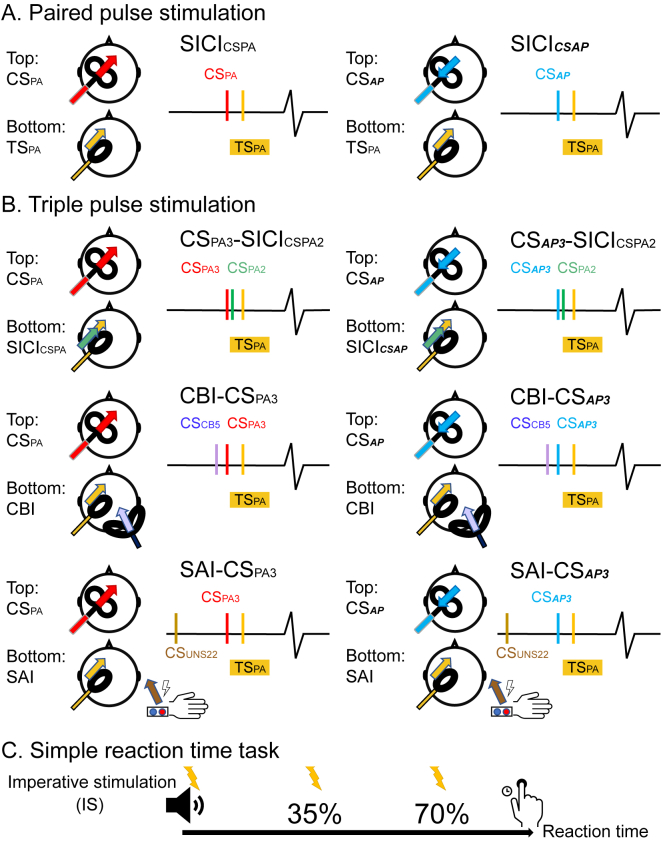
TMS Coil Set-up (A, B) and protocol of simple reaction time task (C). The figure-of-eight coil connecting by current reversing cable was applied to induce anterior-posterior direction conditioning current in paired-pulse stimulation (A) and triple-pulse stimulation (B). There were three triple-pulse stimulation protocols here: triple pulse stimulation with two cortical conditioning, with cerebellar conditioning, and with peripheral sensory afferent input. The direction and colour of arrows indicate the orientation and coil selected to induce currents over the cortex. The images on the left side depict scenarios in which both coils (conditioning stimulus: figure-of-eight coil in red; test stimulus: oval-coil in yellow) delivered posterior-anterior (PA) currents. In triple-pulse stimulation, another conditioning stimulus was applied with the oval-coil (green), cerebellar conditioning stimulus (violet), or peripheral sensory afferent input (brown). On the right, the figure-of-eight coil produces an anterior-posterior (***AP***) current (blue). The bottom figure illustrated the timing giving SICI during a simple reaction time task (C).

As previously reported [25], we arranged the coil so that the side of the oval coil resting on the hotspot overlapped with the junction region of the figure-of-eight coil. The coils were then tied securely to each other with the oval coil under the D50 coil. (see Fig. 1A; also see Supplementary material I and Fig. S1 for methodological details). The motor hot spot was defined as the position on the left motor cortex where supra-threshold PA currents in the oval coil produced the largest and most consistent MEPs in the right FDI muscle. AMTs measured in PA direction (AMT_PA_) were defined separately for both the D50 coil and the oval coil as the minimal intensity to evoke an MEP of more than 200 μV in at least 5 of 10 trials while participants contracted their right FDI muscle by 10% maximum voluntary contraction [26]. AMT for the ***AP*** direction (AMT_***AP***_) was measured in the D50 coil after connecting a current reversing cable. Whenever the text refers to the intensity of a CS, it is sometimes expressed relative to the AMT (PA or ***AP*** as appropriate) of the coil delivering that stimulus and sometimes relative to AMT_PA_ regardless of direction. The text will indicate which was employed.

### 2.5 Short-interval intracortical inhibition (SICI)

We used the short-interval intracortical inhibition (SICI) paradigm, where a sub-motor threshold pulse (conditioning stimulus; CS) has the effect of suppressing the MEP generated by a subsequent supra-motor threshold pulse (test stimulus, TS) delivered a few milliseconds later. To simplify interpretation of the data, the TS (from the oval coil) was always delivered in the PA direction (TS_PA_); the CS stimulus was in either the PA or ***AP*** direction (CS_PA_ or CS_***AP***_), depending on the direction of current in the figure-of-eight conditioning coil (D50) (Fig. 1A). The current reversal required the insertion of a reversing cable such that it was not possible to randomise the CS direction from trial to trial. Instead, blocks of trials were performed with the same CS direction. The intensity of the TS was adjusted to evoke an MEP of approximately 1 mV peak-to-peak in the relaxed right FDI. SICI was calculated as the ratio between the averaged conditioned responses (CS + TS) and the test (TS) MEP amplitudes. In this study, SICI_CSPA_ means SICI consisting of CS_PA_ and TS_PA_, and SICI_***CSAP***_ indicates SICI was composed of CS_***AP***_ and TS_PA_.

### 2.6 Cerebellar-M1 inhibition (CBI)

To evaluate cerebellar-M1 connectivity (CBI), we used the paired-pulse protocol described by Ugawa et al. [27]. The conditioning pulse was delivered over the right cerebellum, 3 cm lateral to the inion, 5 ms prior to a TS_PA_ over M1 (CS_CB5_). The conditioning pulse flowed in the superior direction in the cerebellum, and the intensity of the cerebellar CS was set to −5% maximum stimulator output (MSO) below the brainstem motor threshold (AMT_BA_) [27]. This was measured by stimulating the cerebellum over the inion while subjects contracted their FDI muscle. This threshold was defined as the stimulator output that elicited an MEP of 50 μV in 5 of 10 trials. In 5 participants, there was no response at 80% MSO, which is often perceived as unpleasant. In these cases, the CS_CB5_ intensity was set to 75% MSO [28]. Stimuli were randomized such that there were 15 trials of conditioned responses for every 15 TS tested. CBI was calculated as the ratio of the average MEP amplitudes conditioned by cerebellar stimulation to the average MEP response elicited by TS alone.

### 2.7 Short-latency afferent inhibition (SAI)

To investigate how somatosensory input interacts with SICI, we applied a conditioning peripheral nerve stimulation (DS7A; Digitimer Ltd, Welwyn Garden City, Hers, UK) to the ulnar nerve prior to M1 TMS (CS_UNS_). The inter-stimulus intervals (ISI) between electrical CS and TMS were set according to individual N20 somatosensory-evoked potential latency [29]. We investigated SAI with ISI of ∼22 ms (N20 + 2, CS_UNS22_) inputs using a square-wave pulse width of 200 μs. The intensity of peripheral CS was adjusted to elicit a 0.2 mV FDI M-wave response (i.e. just above motor threshold [30]). Similar to CBI, SAI was quantified as a ratio of the mean amplitude of 15 CS + TS MEPs to 15 TS MEPs.

### 2.8 Protocols

#### 2.8.1 Experiment 1. SICI at ISIs = 2 and 3 ms using different intensities and orientations of CS

This experiment investigated how different current directions and conditioning intensities affected SICI. We recruited 11 healthy subjects (28.8 ± 4.2 years old, 4 male and 7 female). We selected ISIs of 2 ms and 3 ms since previous work had found differences in the effect of PA and ***AP*** conditioning stimuli at these intervals [12]. First, the AMT was measured in both PA (AMT_PA_) and ***AP*** directions (AMT_***AP***_) with the D50 figure-of-eight coil. The intensities used for both CS_PA_ and CS_***AP***_ were 60, 70, 80, 90, 100 and 110% of AMT_PA_. These were supplemented for CS_***AP***_ with the additional intensities of 60, 70, 80, 90, 100 and 110% of AMT_***AP***_. This allowed us to assess the entire profile of SICI_***CSAP***_ from low to high intensity since previous studies only used AMT_***AP***_ when evaluating SICI_***AP***_. The ISI was randomized between blocks of trials in which the six different intensities were intermixed. Thus, each block consisted of a total of 105 pulses: 15 pulses per each CS (6 × 15 = 90) and 15 TS pulses. Since each block of trials lasted up to 7–8 min, we had a team member looking at participants during testing and reminding participants to maintain attention.

#### 2.8.2 Experiment 2. SICI at ISIs = 1, 2, 3 and 5 ms using different orientations of CS

In this experiment, we fixed the intensity of the CS to 90% AMT_PA_ (see the result of experiment 1) in order to explore ISIs over a wider range. This experiment included 15 participants (27.3 ± 4.9 years old, 8 male and 7 female). We performed 15 pulses for each ISI state. Within each block of trials, the CS orientation was constant, but the ISIs were randomized.

#### 2.8.3 Experiments 3, 4 and 5

All of these experiments compared the interaction of SICI using opposite orientations of CS (i.e. CS_***AP***_ and CS_PA_) with other forms of cortical inhibition. However, we had to first decide the ISI and CS intensity to evoke SICI. The results of experiments 1 and 2 showed that SICI evoked with CS_***AP***_ and CS_PA_ had different time courses. But at ISI = 3 ms, they both appeared to produce a similar amount of inhibition. Importantly, both SICI_CSPA3_ and SICI_***CSAP3***_ produced effective inhibition with a CS intensity of 90% AMT_PA_ no matter what the orientation of the CS (see the result of experiments 1 and 2). This raises the question as to whether both current directions activate the same inhibitory system at ISI = 3 ms, even if they do not work at ISI = 2 ms. Thus, in the following experiments, we set the ISI to 3 ms and the CS intensity to 90% AMT_PA_ for both directions of conditioning current (Fig. 1B).

##### 2.8.3.1 Experiment 3. SICI produced by two CS: the interaction of CS_PA_ and CS_*AP*_

Previous triple-pulse TMS studies showed that SICI_CSPA_ could be enhanced by applying a second CS_PA_ a few ms before the conditioning stimulus and that the amount of inhibition is greater than the expected sum of each stimulus alone (i.e. temporal summation [19]). The aim of experiment 3 was to test whether two conditioning stimuli of opposite directions interacted in the same way as two CS_PA_.

We refer to the earliest CS as CS1, and the later CS as CS2, with the TS being the last in the series. CS2 was applied through the oval coil in the PA direction using an ISI = 2 ms (SICI_CSPA2_) prior to the TS (also in the PA direction). The intensity of CS2 was expressed relative to the AMT_PA_ in the oval coil (90% AMT_PA_). CS1 was applied via the D50 coil at an ISI = 3 ms prior to TS. The orientation of current in CS1 was reversed with the reversing cable randomly between blocks (i.e. CS_***AP3***_ and CS_PA3_ at 90%AMT_PA_). In other words, we measured how SICI evoked by CS_PA2_ was affected by the presence of either CS_PA3_ (CS_PA3_-SICI_CSPA2_) or CS_***AP3***_ (CS_***AP3***_-SICI_CSPA2_) (Fig. 1B). Thus, we would compare the effect of interacting SICI evoked by either CS_PA3_ or CS_***AP3***_ with a second CS at 2 ms (CS_PA2_), arguing that if CS_PA3_ and CS_***AP3***_ were both activating the same inhibitory system at ISI = 3 ms, then they would show equal summation with CS_PA2_. Thirteen subjects (27.5 ± 5.0 years old, 8 male and 5 female) were recruited. For each block, 15 trials of each condition were tested. The conditions in experiments 3 and 4 were intermixed within the same recording session. Details are given in Table 1.

**Table 1:** Stimulation conditions in Experiment 3, 4, and 5.

Experiment	Block	Condition
Experiment 3, 4	1	Test stimulation, SICI_CSPA3_, CBI-CS_PA3_
	2	Test stimulation, SICI_***CSAP3***_, CBI-CS_***AP3***_
	3	Test stimulation, CBI
	4	Test stimulation, SICI_CSPA2_, SICI_CSPA3_, CS_PA3_-SICI_CSPA2_
	5	Test stimulation, CS_***AP3***_-SICI_CSPA2_
Experiment 5	6	Test stimulation, SAI
	7	Test stimulation, SICI_CSPA3_, SAI-CS_PA3_
	8	Test stimulation, SICI_***CSAP3***_, SAI-CS_***AP3***_

For experiments 3 and 4, ten participants received blocks 1–5 randomly in a single session. However, this sequence proved time consuming and eleven participants only managed blocks 1, 2, and 3 in a single session. All sixteen individuals contributed data to experiment 4.

Three participants requested to be excused from CBI because of discomfort. They received blocks 2 (but without CBI-CS_***AP3***_), 4, and 5 randomly in a single session. These contributed data to experiment 3 together with the ten participants who had received all 5 blocks (i.e. 13 participants in total).

Fourteen individuals performed experiment 5 with blocks 6–8 intermixed randomly in a single session.

For details of participants' distribution in each experiment, please see supplementary material 5.

##### 2.8.3.2 Experiment 4. Interaction of SICI using either CS_PA_ or CS_*AP*_ with CBI

The interaction between CBI and SICI evoked by CS_PA_ has been reported in a previous publication by Daskalakis and colleagues [20]. In experiment 4, we used the CBI protocol to activate cerebello-thalamo-cortical inputs to M1 and measured the effects on SICI with different conditioning stimulus directions (CS_PA3_ and CS_***AP3***_). The question addressed here is whether CBI interacts differently with SICI evoked by CS_PA3_ or CS_***AP3***_. Sixteen healthy volunteers were recruited (26.5 ± 4.6 years old, 9 male and 7 female). The paradigm was similar to the protocol described by Daskalakis et al. [20] and involved three stimuli. The first stimulus was applied to the cerebellum (CS_CB5_) 5 ms prior to the TS over M1 (CBI). SICI was produced by either CS_PA3_ and CS_***AP3***_. In other words, the triple pulse combinations were CBI-CS_PA3_ and CBI-CS_***AP3***_ (Fig. 1B), and paired pulse combinations were SICI_CSPA3_, SICI_***CSAP3***_ and CBI. Fifteen responses of each condition were averaged and compared. As mentioned in experiment 3, the conditions in experiments 3 and 4 were intermixed within the same recording session. Details are given in Table 1.

##### 2.8.3.3 Experiment 5. Interaction of SICI using either CS_PA_ or CS_*AP*_ with SAI

Since previous work has reported the interaction of SAI with SICI_CSPA_ [[21], [22], [23]], the question here was whether SAI interacts differently with SICI evoked with CS_PA3_ and CS_***AP3***_. Fourteen subjects (27.1 ± 4.9 years old, 7 male and 7 female) participated in this session with a triple-pulse stimulation protocol similar to experiment 4. Here, a peripheral nerve CS was applied 22 ms (CS_UNS22_) prior to the TS over M1. SICI again was either produced by CS_PA3_ or CS_***AP3***_. Thus, conditions in this session included two different combinations of triple pulse stimulation (SAI-CS_PA3_ and SAI-CS_***AP3***_) (Fig. 1B) and paired stimulation (SAI, SICI_CSPA3_ and SICI_***CSAP3***_). In addition, the set of CS_PA3_ and the set of CS_***AP3***_ were tested separately with randomized order of conditions. Each block included 15 trials of each condition. Conditions tested in this experiment are listed in Table 1.

#### 2.8.4 Experiment 6. Changes of SICI in a simple reaction time task

Thirteen healthy participants (25.2 ± 5.5 years old, 7 male and 6 female) were enrolled in this experiment. We probed SICI_CSPA3_ and SICI_***CSAP3***_ in 3 different brain states: at rest, during tonic muscle activation, and while subjects performed a simple reaction time task (SRTT). During tonic activation of muscle, participants were asked to make an isometric (approximately 5% MVC) contraction of FDI (in one block) or abductor digiti minimi muscle (ADM, in another block) muscle while SICI was measured. In SRTT, there was no warning cue; the imperative (“go”) stimulus (IS) was an auditory tone burst (100 ms) of 500 Hz given randomly every 5 ± 0.5 s. Individuals had to tap their index finger as quickly as possible on hearing the tone. SICI was tested at 3 time points in the reaction time period: at the cue, the early stage of reaction time (RT_35%_), the late stage of reaction time (RT_70%_) (Fig. 1C). RT timings were adjusted to each individual's mean reaction time, taken from a practice session consisting of 20 trials.

The test pulse intensity was set to elicit a ∼1 mV peak-to-peak amplitude for all conditions (rest, tonic and SRTT). Both conditioning pulse directions (CS_PA_ and CS_***AP***_) were set to 90% AMT_PA_ and the ISI = 3 ms. Throughout all experimental conditions, we recorded 20 test pulses and 20 CS trials. For the SRTT, participants performed the task twice for each muscle, and a total of 140 trials were performed (120 trials with TMS; 20 trials with just the auditory cue).

### 2.9 Data analysis and statistics

EMG recordings were analyzed using Signal software version 7.01 (Cambridge Electronic Design Ltd., Cambridge, UK). The MEPs amplitudes from both unconditioned and conditioned MEPs were considered for analysis. As in previous studies [[31], [32], [33], [34]], we excluded extreme MEP amplitudes in which responses were more than 1.5 times above the third quartile or below the first quartile of the interquartile range. Trials were also excluded if ongoing muscle activity was detected (EMG signal >50 μV within 100 ms before TMS pulse) [34]. The total number of discarded MEPs amounted to 4.9% of all recordings in this study. In experiment 1, the SICI data is plotted against both relative (% of AMT) and absolute intensities (% of Maximum Stimulator Output, MSO) of the CS. Absolute intensities of 70% AMT_***AP***_ (48 ± 7.6%) and 100% AMT_***PA***_ (48 ± 9.1%) did not significantly differ (48% MSO) (t = 0.052, df = 10, p = 0.959). SPSS version 22 (IBM Co., US) was used for all statistical analyses.

All measures of inhibition in this paper used the conditioned/test MEP amplitude ratio as the variable of interest, where values lower than 1 indicate inhibition. In experiment 1, we compared the effect of CS intensity on SICI_CSPA_ and SICI_***CSAP***_ separately at each ISI (i.e. 2 or 3 ms) with a two-way repeated-measures ANOVA (RM-ANOVA) with factors “Intensity” (60%, 70%, 80%, 90%,100%, and 110% AMT_PA_) and “Orientation” (CS_PA_ and CS_***AP***_). The data from this experiment were also used to select the optimal CS intensity to use at ISI = 3 ms in all subsequent experiments. To do this, additional one-tailed t-tests (to avoid type II error) were used to compare the amount of inhibition (SICI) at each CS intensity with baseline (ratio = 1) (see results Experiment 2). In four cases (SICI_***CSAP3***_ with 100% and 110% AMT_***AP***_, SICI_***CSAP3***_ with 60% AMT_PA_, SICI_***CSAP3***_ with 110%AMT_PA_), a Shapiro-Wilk test showed a violation of normality. In these instances, we used a one-tailed Wilcoxon signed-rank test in experiment 1.

In experiment 2, a two-way RM-ANOVA with factors “Orientation” (CS_PA_ and CS_***AP***_) and “ISI” (1, 2, 3, and 5 ms) was used to compare SICI_CSPA_ and SICI_***CSAP***_. Here one-tailed t-tests were done again to examine the effectivity of inhibition, and a Shapiro-Wilk test was done for checking normality.

In experiments 3, 4 and 5, we compared the amount of inhibition produced by SICI_***CSAP3***_ and SICI_CSPA3_ alone with that seen during the presence of other forms of inhibition (SICI_CSPA2_, CBI, SAI). To do so, we used two separate one-way RM-ANOVAs and a two-way RM-ANOVA in each experiment. For example, in experiment 4, where CBI interacted with SICI, we first performed 2 separate one-way RM-ANOVAs: one compared CBI, SICI_***CSAP3***_ and CBI-CS_***AP3***_, and the other compared CBI, SICI_CSPA3_, and CBI-CS_PA3_. The question here was whether the inhibition produced by CBI combined with SICI differed from that produced by SICI alone. A two-way ANOVA determined whether CBI interacted with SICI_CSPA3_ and SICI_***CSAP3***_ in different ways. It had “Orientation” (CS_***AP***_ and CS_PA_) and “Condition” (SICI alone (SICI_CSPA3_ and SICI_***CSAP3***_) and SICI in the presence of CBI (CBI-CS_PA3_ and CBI-CS_***AP3***_) as main factors. Therefore, the structure to access interaction in the two-way RM-ANOVA was (SICI_CSPA3_, CBI-CS_PA3_) x (SICI_***CSAP3***_, CBI-CS_***AP3***_). These correspond to the 4 right-hand columns of Fig. 5. Additionally, in order to check the consistency of our data with those of previous triple pulse studies [20,21,23], changes in SICI during CBI or SAI were calculated using the method proposed by Daskalakis and co-workers [20] (See Supplementary material 6 for details). Daskalakis et al. calculated the change in SICI_CSPA2_ in the presence of CBI as by [amplitude of MEP conditioned by CBI-CS_PA2_]/[amplitude of MEP by CBI only]. We used the same method to compute the change in SICI_CSPA3_ (when CBI or SAI was presented). Normality was tested by the Shapiro-Wilk test. Paired t-tests were applied to compare SICI_CSPA3_ with the change in SICI_CSPA3_ when CBI or SAI was also performed.

In experiment 6, two separate two-way RM-ANOVAs with “Brain state” (Rest, Tonic muscle contraction) and “Orientation” (SICI_CSPA3_ and SICI_***CSAP3***_) as main factors were used to investigate changes in SICI in different brain states in each muscle (FDI and ADM). Then, we used a further two-way RM-ANOVA with factors “Orientation” (SICI_CSPA3_ and SICI_***CSAP3***_) and “Time” (on the cue, RT_35%_ and RT_70%_) to assess changes in SICI during the preparation of movement, separately in each muscle (FDI and ADM).

Mauchly's test was used to check sphericity in all RM-ANOVAs, and Greenhouse-Geisser correction was used for non-sphericity conditions. Although RM-ANOVA is quite robust to violations of normality, we nevertheless checked the normality of residuals with Quantile-Quantile Plots (Q-Q plots) in each experiment. There were no obvious violations of normality in these datasets. The critical value of significance was 0.05 in all statistics. Post hoc pairwise comparisons employed a Bonferroni correction.

## 3 Results

### 3.1 Experiment 1

**Fig. 2:**
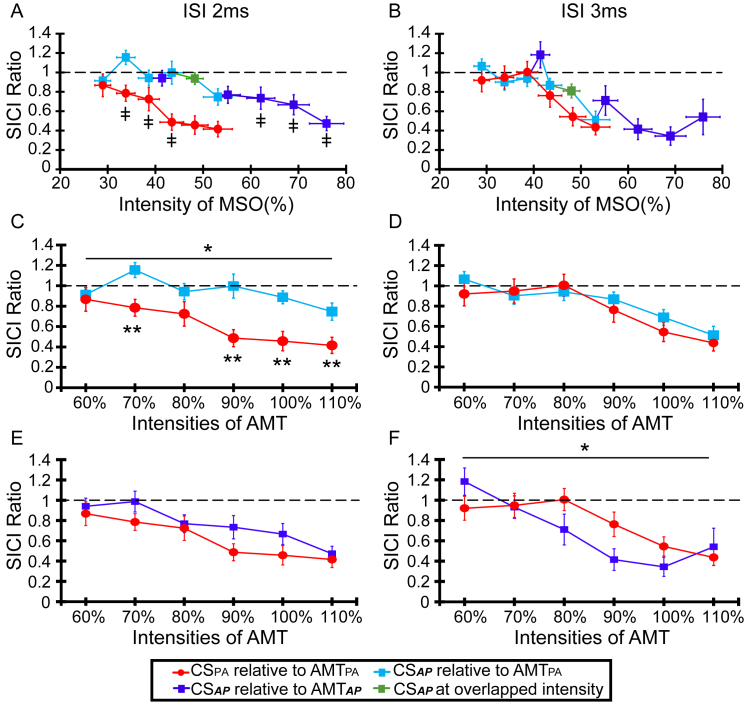
SICI at 2 and 3 ms ISI: effect of different conditioning stimulus intensities for CS_*AP*_ and CS_PA_. The left column (A, C, E) depicts SICI at ISI = 2 ms and in the right column (B, D, F) SICI at ISI = 3 ms. Panels in the top row (A, B) plot the CS intensity in percent MSO; panels in the middle row (C, D) plot CS intensity in percent of AMT_PA_; panels (E, F) in the bottom raw plot CS intensity in percent of direction-appropriate AMT (i.e. AMT_***AP***_ for CS_***AP***_, AMT_PA_ for CA_PA_). When intensities of conditioning stimulation were given based on AMT_PA_, panel D shows that at ISI = 3 ms, both CS_***AP***_ (light blue) and CS_PA_ (red) have approximately the same threshold (i.e. 90% AMT_PA_). In contrast, at ISI = 2 ms (panel C), inhibition is evident only with CS_PA_ but not with CS_***AP***_. Because of the lack of SICI over these intensities with CS_***AP***_, an additional run was performed in which the intensity of CS_***AP***_ was expressed in percent AMT_***AP***_. When intensities of conditioning stimulation were giving based on the AMT of the direction of conditioning stimulation (i.e. CS_***AP***_ by AMT_***AP***_), panel F reveals that SICI_***CSAP3***_ by AMT_***AP***_ (deep blue) is significantly different from SICI_CSPA3_ by AMT_PA_ (red). In contrast, panel E shows that SICI_***CSAP2***_ by AMT_***AP***_ (deep blue) is not different from SICI_CSPA2_ by AMT_PA_ (red). The data from both these runs are combined in panels A and B, in which the intensity of the conditioning stimuli is expressed in %MSO in order to plot all data on the same x-axis. Because AMT varies (in terms of %MSO) across individuals, the data are plotted with x-error bars. In each graph, the data points in red and light blue replot the data in panels C and D; the dark blue points and line plot data from the additional runs at higher intensities of CS_***AP***_. Green points are data from points in the two runs of CS_***AP***_ in which the absolute intensities of CS were the same, and thus the data was combined. The graphs show that at ISI = 3 ms, the threshold for producing SICI is approximately the same for both directions of CS. However, at ISI = 2 ms, SICI was evident only with high intensities of CS_***AP***_. The single asterisk indicates the significant (p < 0.05) “Orientation” X “Intensity” interaction in a two-way RM-ANOVA; double asterisks indicate intensities with a significant difference (post hoc pairwise comparison) in the amount of inhibition produced by CS_PA_ and CS_***AP***_. (For interpretation of the references to colour in this figure legend, the reader is referred to the web version of this article).

This experiment investigated how SICI at ISIs = 2 and 3 ms varied with the intensity and orientation of the CS. Fig. 2A and Fig. 2B gives an overall picture of the results. CS intensities are given in MSO in order to display all data using the same x-axis; the two curves on each graph display effects of the two orientations of CS. Note that x-error bars (±SEM) are necessary since in the actual experimental sessions, individual CS intensities were expressed relative to AMT, which differed in terms of MSO for each person. In addition, since there was no obvious SICI at an ISI = 2 ms with CS_***AP***_, we extended the range of intensities for this orientation to 60–110% AMT_***AP***_. These two ranges are indicated in the graphs by dark and light symbols, respectively. The absolute intensities of the conditioning stimuli may appear to be quite high; this is because the conditioning coil was placed over the test coil which increased the distance between the scalp surface and the conditioning (D50) coil.

**Fig. 3:**
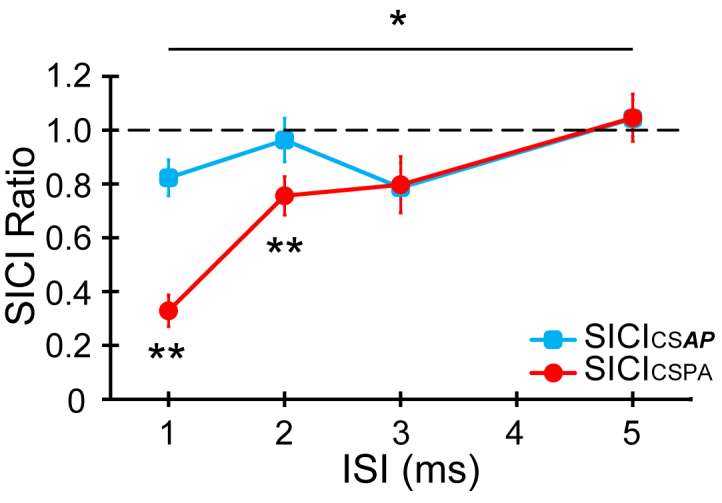
Comparison of SICI produced by the same intensity of CS_PA_ and CS_*AP*_ over a range of ISIs. The x-axis depicts the ISI selected between the CS and TS pulses. Both CS_PA_ (red) and CS_***AP***_ (blue) intensities were set to 90% AMT_PA_. We found significant differences between the two different CS at ISIs = 1 ms and 2 ms but not at other intervals. Asterisks indicate a significant orientation × ISI interaction in a two-way ANOVA, p < 0.05. Double asterisks indicate p < 0.05 in post hoc pairwise comparison between the different orientations at each ISI. (For interpretation of the references to colour in this figure legend, the reader is referred to the web version of this article).

This is illustrated more clearly in Fig. 2C and D, which directly compare CS intensities in those blocks where intensity was expressed relative to AMT_PA_ (for both current directions). The x-axis plots CS intensity as a percent of AMT_PA_. At ISI = 3 ms (Fig. 2D), the recruitment of SICI is similar for both directions of CS. In contrast, at ISI = 2 ms (Fig. 2C), CS_***AP***_ evokes very little SICI at any intensity whilst it is clear for CS_PA_. For completeness, Fig. 2E and F plot the comparison between CS_PA_ and CS_**AP**_ in terms of the AMT of each direction of CS. Fig. 2E (ISI = 2 ms) shows that the threshold for producing SICI (relative to AMT of the CS) is the same for each CS direction. However, the situation at ISI = 3 ms is quite different. When expressed in terms of the direction-appropriate AMT, the threshold for SICI with CS_***AP***_ was lower than that for CS_PA_.

The impression given by the graphs was borne out in the statistical analysis. In Fig. 2C, a two-way RM-ANOVA between CS_PA_ and CS_***AP***_ at ISI = 2 ms and CS intensities expressed for both orientations in terms of AMT_PA_, revealed a main effect of “Orientation” (F_1,10_ = 12.119, p = 0.006), and a significant “Orientation x Intensity” interaction (F_5,50_ = 3.113, p = 0.016). Post hoc pairwise comparison showed that this was due to the fact that SICI_CSPA2_ was stronger than SICI_***CSAP2***_ (p = 0.02 at 70%, p = 0.006 at 90%, p = 0.002 at 100%, and p = 0.013 at 110%AMT) (Fig. 2C). In contrast, no differences were found between CS directions at ISI = 3 ms (Orientation: F_1,10_ = 1.000, p = 0.341; Orientation x Intensity: F_5,50_ = 0.509, p = 0.768) (Fig. 2D). In Fig. 2E (ISI = 2 ms), there were no main or interaction effects. In contrast, in Fig. 2F (ISI = 3 ms), there was a significant “Orientation x Intensity” interaction (F_5,50_ = 3.690, p = 0.006), which was due to the fact that CS_**AP**_ evoked inhibition at a lower threshold relative to the AMT of the CS than CS_PA_.

To summarise, at ISI = 3 ms, the threshold for recruiting SICI was the same with both CS orientations, whereas at ISI = 2 ms, the absolute threshold was much higher for CS_***AP***_ than CS_PA_, although it was approximately the same in terms of the relative AMT for each direction.

### 3.2 Experiment 2

In this experiment, the intensity of the CS was the same for both directions (90% AMT_PA_), while we tested ISIs shorter and longer than in experiment 1. The data in Fig. 2D show that 90% AMT_PA_ was the lowest intensity that produced significant inhibition (one-tailed *t*-test compared to baseline) with both orientations of CS (t = −1.84, df = 10, p = 0.048 for SICI_***CSAP3***_ and t = −1.97, df = 10, p = 0.039 in SICI_CSPA3_). At ISIs = 2, 3 ms, the results are very similar to those in experiment 1: both directions of CS produced the same amount of SICI at ISI = 3 ms, whereas SICI was absent at ISI = 2 ms for CS_***AP***_, whilst it was clear for CS_PA_. An even greater difference was seen at ISI = 1 ms, where there was strong inhibition for CS_PA_ but much less for CS_***AP***_. Neither direction of CS produced inhibition at ISI = 5 ms.

The two-way RM-ANOVA revealed a significant “Orientation x ISI” interaction (F_3,42_ = 13.902, p < 0.001); post hoc pairwise comparison showed a stronger SICI with CS_PA_ than CS_***AP***_ at 1 ms (p < 0.001) and 2 ms (p = 0.020). Compared with baseline, there was a small amount of inhibition with CS_**AP1**_ (one-sample *t*-test, df = 14, t = −2.623, p = 0.01) but no detectable inhibition with CS_***AP2***_. In summary, the results confirm those of experiment 1 (also see supplementary material II and Fig. S2) and extend the differences in CS direction to ISI = 1 ms.

### 3.3 Experiment 3: Interaction between SICI_CSPA_ and SICI_*CSAP*_

**Fig. 4:**
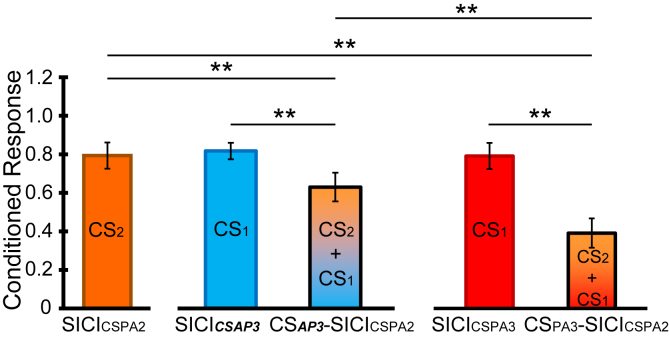
Interaction between CS_PA_ and CS_*AP*__*. This experiment was designed to compare the interaction of CSPA3 and CS**AP3** with CSPA2. The left panel shows the amount of SICI produced by CSPA2 alone. The middle panel shows the amount of SICI produced by CS**AP3** alone and the amount produced by the combination of CS**AP3** and SICICSPA2. The right panel shows the amount of SICI produced by CSPA3 alone and the amount produced by the combination of CS**AP3** and SICICSPA2. Both combinations produce more inhibition than either conditioning stimulus alone, but the effect is much larger with CSPA3-SICICSPA2 than with CS**AP3**-SICICSPA2. Double asterisks indicate p < 0.05 in post hoc pairwise comparison. CS1 indicates the earlier CS, and CS2 means the later CS.*_

Fig. 4 shows that when given alone, all three CS (CS_PA3_, CS_***AP3***_, and CS_PA2_) evoked approximately the same amount of SICI. However, CS_PA3_-SICI_CSPA2_ evoked much greater inhibition than CS_***AP3***_-SICI_CSPA2_. One way of comparing these values is to ask how much SICI we might expect to observe from combining the effects of two CS. The level of SICI evoked by CS_***AP3***_ and CS_PA2_ alone was 0.82 and 0.79, respectively. Thus if the effects were independent, we should expect that the result of combining them should be 0.82 ∗ 0.79 = 0.65. This prediction is similar to the observed value of 0.63 when CS_***AP3***_ and CS_PA2_ were combined experimentally (i.e. CS_***AP3***_-SICI_CSPA2_). This contrasts with the combination of CS_PA2_ and CS_PA3_ (i.e. CS_PA3_-SICI_CSPA2_). This combination produced SICI = 0.39 which was much more powerful than expected from independently combining SICI_CSPA3_ (0.79) and SICI_CSPA2_ (0.79 ∗ 0.79 = 0.62). Thus, rather than acting independently, CS_PA2_ and CS_PA3_ show temporal facilitation and produce more SICI than expected.

**Fig. 5:**
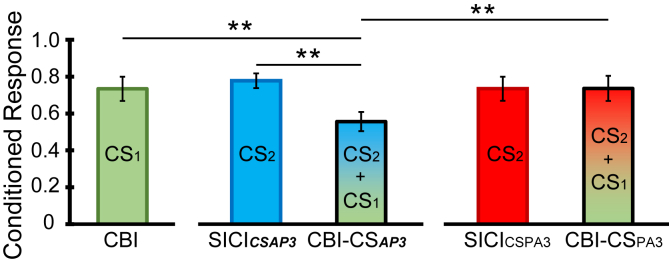
Interaction between SICI and CBI. The left panel depicts the effect of cerebellar stimulation alone (CBI: ISI = 5 ms) compared to the responses elicited when combined with CS_***AP3***_ (middle-panel) or CS_PA3_ (right-panel). The combination of CBI and CS_***AP3***_ produced more inhibition than either conditioning stimulus alone, whereas the combination of CBI and CS_PA3_ produced approximately the same amount of inhibition as either conditioning stimulus alone. Double asterisks indicate p < 0.05 in post hoc pairwise comparison. CS1 indicates the earlier CS, and CS2 means the later CS.

In the statistical analysis, we compared the level of inhibition produced by CS_PA3_-SICI_CSPA2_, SICI_CSPA3_, and SICI_CSPA2_. A one-way RM-ANOVA revealed a significant, global difference among them (F_2,24_ = 21.387, p < 0.001). Post hoc pairwise comparison showed that CS_PA3_-SICI_CSPA2_ was significantly different from both SICI_CSPA3_ (p < 0.001) and SICI_CSPA2_ (p = 0.001). Similarly, another one-way RM-ANOVA on the level of SICI produced by CS_***AP3***_-SICI_PA2_, SICI_***CSAP3***_, and SICI_CSPA2_ revealed a significant difference among them (F_2,24_ = 4.668, p = 0.019). Post hoc pairwise comparison showed that CS_***AP3***_-SICI_PA2_ was different from both SICI_***CSAP3***_ (p = 0.039) and SICI_CSPA2_ (p = 0.035). Crucially, the two-way RM-ANOVA with “Orientation” (CS_PA_ and CS_***AP***_) and “Condition” (SICI at ISI of 3 ms alone (SICI_CSPA3_ and SICI_***CSAP3***_) and SICI at ISI of 3 ms in the presence of SICI_CSPA2_ (CS_PA3_-SICI_CSPA2_ and CS_***AP3***_-SICI_CSPA2_)) as main factors revealed an “Orientation x Condition” interaction (F_1,12_ = 4.943, p = 0.046), which post hoc pairwise comparison showed was due to the fact that CS_PA3_-SICI_CSPA2_ induced stronger cortical inhibition than CS_***AP3***_-SICI_PA2_ (p = 0.004).

### 3.4 Experiment 4. Interaction of SICI using either CS_PA_ or CS_*AP*_ with CBI

Since the results in experiment 3 suggested that there are differences in the inhibition produced by ***AP*** and PA conditioning stimuli, we next tested whether these would interact in different ways with another input to M1.

Fig. 5 shows that CBI produced approximately the same amount of inhibition as SICI with CS_***AP3***_ or CS_PA3_. CBI-CS_PA3_ yielded no greater inhibition than either alone, whereas CBI-CS_**AP3**_ produced more inhibition than either stimulus alone.

In the statistical analysis, we compared the level of inhibition evoked by CBI, SICI_***CSAP3***_ and CBI-CS_***AP3***_. A one-way RM-ANOVA with Greenhouse-Geisser correction revealed a significant difference between them (F_1.480,22.199_ = 5.902, p = 0.014). Post hoc pairwise comparison showed that CBI-CS_***AP3***_ was significantly different from both SICI_***CSAP3***_ (p = 0.003) and CBI (p = 0.027). However, there were no significant differences in another one-way ANOVA including CBI, SICI_CSPA3_ and CBI-CS_PA3_ (F_1.459,21.883_ = 0.001, p = 0.996). Crucially, the two-way RM-ANOVA with “Orientation” (CS_PA_ and CS_***AP***_) and “Condition” (SICI alone and SICI in the presence of CBI) as main factors revealed an “Orientation x Condition” interaction (F_1,15_ = 14.756, p = 0.002), which post hoc pairwise comparison showed was due to the fact that CBI-CS_***AP3***_ produced more inhibition than CBI-CS_PA3_ (p = 0.001). Interestingly, we also found that the calculated expected sum of CBI-CS_***AP3***_ (i.e. [CBI (0.73)] ∗ [SICI_***CSAP3***_ (0.78)] = 0.58) was similar to the observed value (0.56).

### 3.5 Experiment 5. Interaction of SICI using either CS_PA_ or CS_*AP*_ with SAI

As in experiment 4, we next examined how sensory afferent inputs to the motor cortex interact with SICI produced by different directions of CS.

**Fig. 6:**
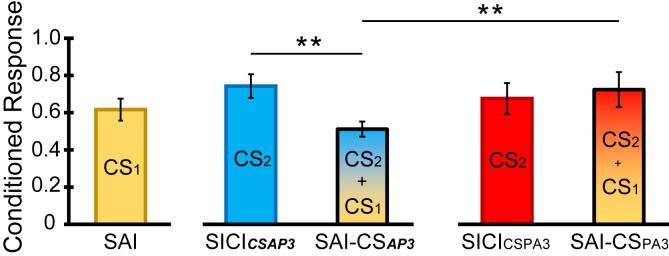
Interaction of SICI with SAI. The left panel shows the MEP conditioned by ulnar nerve stimulation input (SAI; ISI = 22 ms). The middle panel represents SICI obtained with CS_***AP3***_, either alone or combined with ulnar nerve stimulation (SAI-CS_***AP3***_). The right panel plots SICI induced by CS_PA3_ alone and combined with ulnar nerve stimulation (SAI-CS_PA3_). The combination SAI-CS_***AP3***_ produced more inhibition than either conditioning stimulus alone, whereas SAI-CS_PA3_ produced approximately the same amount of inhibition as each conditioning stimulus alone. Double asterisks indicate p < 0.05 in post hoc pairwise comparison. CS1 indicates the earlier CS, and CS2 means the later CS.

Fig. 6 shows that SAI produced approximately the same amount of inhibition as SICI with CS_***AP3***_ or CS_PA3_. SAI-CS_PA3_ yielded no greater inhibition than either alone, whereas SAI-CS_***AP3***_ appears to produce more inhibition than SICI_***CSAP3***_.

In the statistical analysis, we compared the level of inhibition evoked by SAI, SICI_***CSAP3***_ and SAI-CS_***AP3***_. The one-way RM-ANOVA revealed a significant difference among them (F_2,26_ = 6.920, p = 0.004). Post hoc pairwise comparison showed that SAI-CS_***AP3***_ was significantly different from SICI_***CSAP3***_ (p = 0.01) but not different to SAI alone (p = 0.264). However, there were no significant differences in the one-way RM-ANOVA comparing SAI, SICI_CSPA3_ and SAI-CS_PA3_ (F_2,26_ = 1.251, p = 0.303). Crucially, the two-way RM-ANOVA with “Orientation” (CS_PA_ and CS_***AP***_) and “Condition” (SICI alone and SICI in the presence of SAI) as main factors revealed an “Orientation x Condition” interaction (F_1,13_ = 15.561, p = 0.002), which post hoc pairwise comparison showed was due to the fact that SAI-CS_***AP3***_ produced more inhibition than SAI-CS_PA3_ (p = 0.001). Again, the expected combination of SAI-CS_***AP3***_ (i.e. [SAI (0.62)] x [SICI_***CSAP3***_ (0.74)] = 0.46) was not far from the observed value of 0.51.

**Fig. 7:**
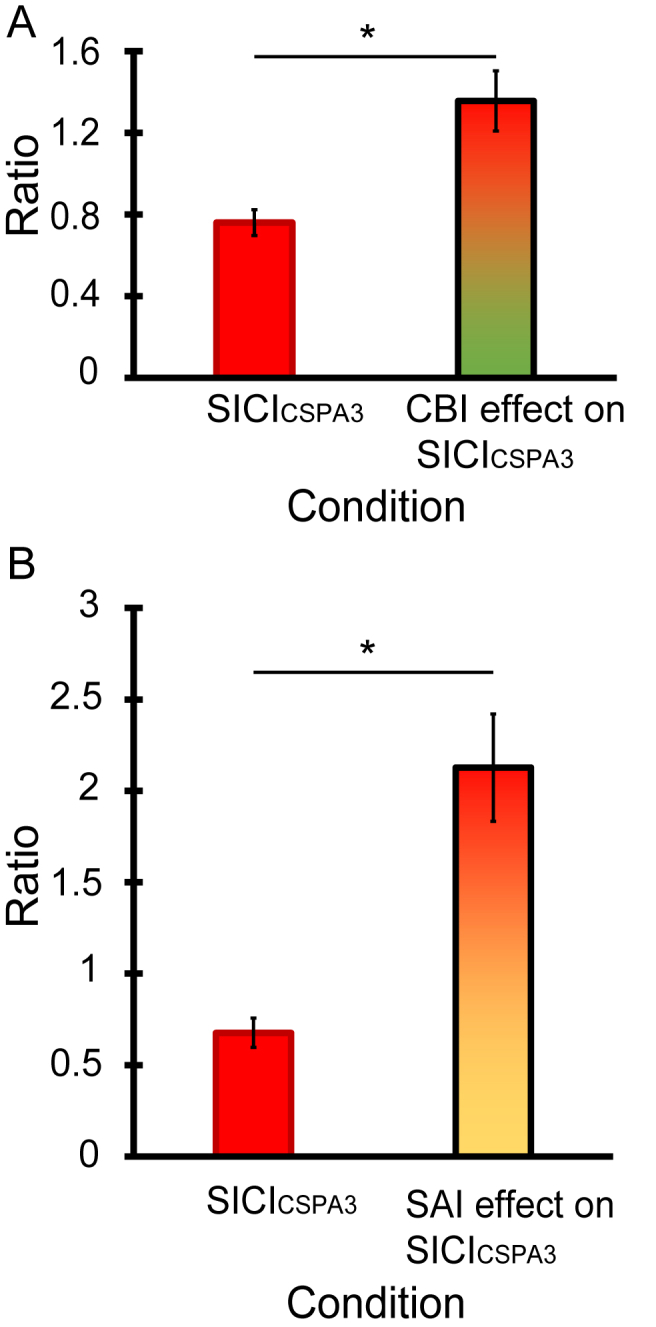
Change of SICI in triple pulses stimulation. Upper panel shows SICI_CSPA3_ and the change of SICI_CSPA3_ in CBI-CS_PA3_ (A). SICI_CSPA3_ was suppressed significantly when CBI was presented in the same trial. Lower panel plots SICI_CSPA3_ and the change of SICI_CSPA3_ when presented with SAI-CS_PA3_ (B). Again, SICI_CSPA3_ was suppressed significantly when SAI was presented at the same trial. Asterisks indicate p < 0.05 in paired *t*-test.

Lastly, the change in SICI_CSPA3_ when CBI was performed was calculated (1.36 ± 0.59). A paired *t*-test revealed that CBI suppressed SICI_CSPA3_ significantly (t = −4.128, df = 15, p = 0.001, Fig. 7A). This result is consistent with Daskalakis et al. [20]. Suppression of SICI also occurred when this was tested together with SAI (2.13 ± 0.29): A paired *t*-test revealed SICI_CSPA3_ was significantly facilitated in the presence of SAI (t = −4.441, df = 13, p = 0.001, Fig. 7B). These results are in agreement with those of Udupa and co-workers [23].

### 3.6 Experiment 6. Changes in SICI in a simple reaction time task

**Fig. 8:**
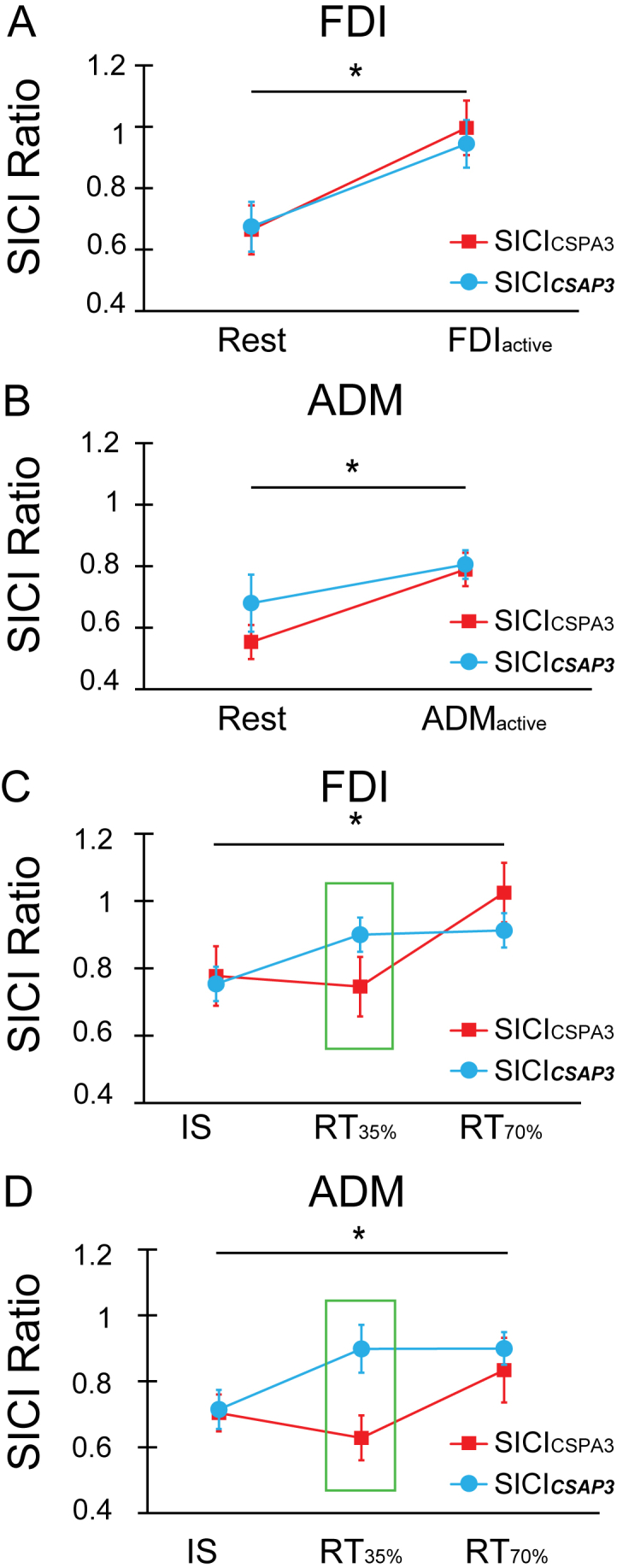
Results from experiment 6. SICI_CSPA3_ and SICI_***CSAP3***_ in both FDI (A) or ADM (B) were equally effective when measured at rest or during tonic contraction. However, there was significantly less SICI during tonic movement than at rest in both muscles. Panels C and D show how SICI changed during the SRTT (C, D). Compared to MEPs evoked at the time of the imperative stimulus (auditory tone), SICI became less effective when probed during the reaction period, although the time course differed between CS_PA_ and CS_***AP***_. SICI obtained with CS_***AP***_ decreased earlier (RT_35%_) than SICI tested with CS_PA_, which only declined at RT_70%_ regardless of whether FDI (C) or ADM (D) were involved in the task. This result suggests that at least two inhibitory networks are involved in controlling action responses, each showing specific temporal dynamics. Asterisks indicate significant main effects of “Brain state” (panels A and B) and significant “Orientation x time” interactions (panels C and D) (p < 0.05). The green box in panels C and D indicate significant differences in post hoc pairwise comparisons (p < 0.05).

For each muscle, we first compared SICI across rest and tonic activation states when probed with different conditioning currents. In the FDI muscle, a two-way RM-ANOVA with “Brain state” and “Orientation” as main factors revealed that there was no effect of “Orientation” (F_1,12_ = 0.155, p = 0.701) or “Orientation x Brain state” interaction (F_1,12_ = 0.412, p = 0.201). However, there was a significant main effect of “Brain state” (F_1,12_ = 9.492, p = 0.010), indicating that contraction reduced SICI. Similar results were seen in ADM. A two-way RM-ANOVA with “Brain state” and “Orientation” as main factors revealed there was no effect of “Orientation” (F_1,12_ = 3.336, p = 0.093) or “Orientation x Brain state” interaction (F_1,12_ = 1.929, p = 0.190), but there was a significant effect of “Brain state” (F_1,12_ = 5.528, p = 0.037) (Fig. 8A and B). In other words, we found no evidence that SICI_CSPA3_ and SICI_***CSAP3***_ respond differently during rest or tonic activation.

The results were different when we investigated how SICI changed during the reaction period of an SRTT. Two-way RM-ANOVAs with “Orientation” and “Time” as main factors revealed a significant “Orientation x Time” interaction in both muscles (FDI: F_2,24_ = 3.675, p = 0.041; ADM: F_2,24_ = 5.670, p = 0.001). Post hoc pairwise comparison revealed that SICI_***CSAP3***_ decreased much earlier during the reaction time (RT_35%_) than SICI_CSPA3_ (FDI: p = 0.035; ADM: p = 0.005) (Fig. 8C and D). The amplitude of the test MEP within blocks of SICI_CSPA3_ did not differ from SICI_***CSAP3***_ in equivalent blocks (see supplementary material III and Fig. S3).

## 4 Discussion

The present experiments explored the effect of reversing the direction of the conditioning stimulus (CS) on short-interval intracortical inhibition (SICI) at interstimulus intervals (ISIs) of 1–5 ms using a range of CS intensities. Conventionally, SICI is produced using a sub-motor threshold posterior-anterior (PA) conditioning stimulus (CS_PA_). The present results suggest that an anterior-posterior conditioning stimulus (CS_***AP***_) of the same intensity can activate a different, and perhaps even independent, inhibitory circuit. We discuss the evidence for this conclusion in the following paragraphs.

### 4.1 SICI with different orientations of CS

Using different coils for the conditioning and test stimuli allows independent adjustment of the direction of CS and TS. This allowed us to maintain a conventional PA test stimulus while reversing the direction of the conditioning stimulus. Most previous investigations [12,14,34,35] have been limited to a single coil, which means SICI can only be assessed with conditioning and test currents applied in the same direction (i.e. ***AP*** to ***AP***; PA to PA). The problem with this is that ***AP*** and PA test stimuli evoke different combinations of I-waves which are differentially sensitive to SICI [14], which confounds interpretation of any differences in the effect of the CS. In addition, some authors (e.g. Hanajima et al. (1998) [14]) have also conducted experiments during slight muscle contraction rather than at rest, which also affects the recruitment of I-waves. In the present experiments, we ensured stability of I-wave recruitment by always using a PA test stimulus in participants at rest; we only changed the orientation of the CS pulse.

A potential disadvantage of this method is that one coil (conditioning coil in the present experiments) rests on top of the other coil, which means that there is an additional distance from the scalp surface. This will reduce its focality and result in activation of a larger cortical area than if the coil were placed in contact with the scalp. However, this is unlikely to have had an important influence on the results since in the past SICI has been tested with a large number of different coil types of varying focality with little noticeable difference in outcome [18,25].

### 4.2 Inhibition at interstimulus intervals of 2 ms and 3 ms

Experiment 1 showed that at 3 ms, the absolute threshold for evoking SICI was approximately the same for both directions of CS (CS_PA_ and CS_***AP***_). This is perhaps unexpected since we usually express the intensity of a CS relative to its own AMT. Nevertheless, it is similar to the conclusion of Ziemann et al. [18], who found that a CS with latero-medial orientation produced the same amount of SICI as CS_PA_ at ISI = 3 ms even though the CS intensity was the same in each direction. In contrast, at 2 ms, CS_***AP***_ was much less effective than (conventional) CS_PA_. Hanajima [12] had also found that CS_***AP***_ was less effective in evoking inhibition at 2 ms than 3 ms, although a direct comparison with the present data is difficult because both CS and TS were in the ***AP*** direction.

The difference in the time course of SICI produced by CS_PA_ and CS_***AP***_ could be because CS_***AP***_ has a delayed onset. The onset of MEPs evoked with a single ***AP*** pulse (***AP***-MEPs) is usually 2–3 ms later than those evoked with a PA pulse (PA-MEPs), so maybe the onset of SICI evoked with CS_***AP***_ is also delayed and begins at 3 ms. However, while this accounts for the difference in latency, it does not explain why the thresholds for CS_***AP***_ and CS_PA_ are approximately equal whereas the threshold for ***AP***-MEPs is higher than for PA-MEPs. The answers may depend on precisely which population of neurons is activated by ***AP*** and PA pulses and their relative locations.

Finally, it is interesting to note that SICI could be evoked at 2 ms with high-intensity CS_***AP***_ that was approximately equal to 90% of the direction-appropriate AMT. This is the same relative intensity required when using a (conventional) CS_PA_. However, it is quite different to the behaviour at ISI = 3 ms, where the intensity of CS_***AP***_ can be much lower than 90%AMT_***AP***_ (also see Supplementary material 7 and Fig. S4). This implies that the circuits involved in SICI at these two intervals are different, at least when using CS_***AP***_.

### 4.3 Inhibition at 1 ms

Fig. 3 not only replicates the observation that SICI_CSPA_ is more effective than SICI_***CSAP***_ at 2 ms but also shows that this is true at 1 ms, even though both directions are equally effective at 3 ms. The origin of SICI at 1 ms is debated. Some authors have suggested that it is due to a synaptic mechanism [16,36,37], whereas others attribute it to axonal refractoriness since CS recruits some axons normally recruited by the TS [25]. We do not know which axons these are, but the assumption is that they provide excitatory input that drives corticospinal discharge and the MEP. In the present result, both SICI_CSPA1_ and SICI_***CSAP1***_ produced effective inhibition, but SICI_CSPA1_ was more effective than SICI_***CSAP1***_. Since MEPs were evoked with a PA test stimulus, CS_PA_ might activate some excitatory inputs that then become refractory to the TS_PA_, resulting in a smaller conditioned response. In contrast, CS_***AP***_ would be unable to activate them since their threshold is much higher for the ***AP*** current. Therefore, it is possible that both theories of SICI at 1 ms contribute to SICI_CSPA1_ but that axonal refractoriness is the predominant mechanism. At first sight, our data seem to differ from those of Hanajima et al. [12] since they reported *good* inhibition at 1 ms. However, in those experiments, *both* the CS and TS were in the ***AP*** direction and would therefore activate the same set of axons involved in producing the test MEP, again consistent with the idea that inhibition at 1 ms is due, at least partially, to axonal refractoriness.

### 4.4 Interaction of separate cortical inhibitory networks

At 3 ms, both directions of CS evoke a similar amount of SICI and have the same (absolute) threshold. The simplest explanation is that they both activate the same set of inhibitory interneurons, which are insensitive to the direction of the TMS pulse. Experiments 3–5 were conducted to test this. The rationale was that if CS_***AP***_ and CS_PA_ activated the same set of inhibitory neurons, then they would interact with other forms of inhibition in the same way. The design of these experiments was less complex than the classic studies of interactions between SICI and SAI or CBI [20,21,23], which explored a range of conditioning and test intensities, as well as interstimulus intervals. The question here was simply whether SICI evoked with CS of different directions, but otherwise of the same intensity, interstimulus interval, and depth of inhibition interacts in the same way with SICI itself, CBI and SAI.

Previous work showed that combining two (conventional) subthreshold CS_PA_ could produce a SICI-like effect if the interval between them was 1–5 ms [19]. The authors suggested that the conditioning stimuli activate excitatory synaptic input to inhibitory neurons [21]. Each CS on its own might fail to generate an excitatory postsynaptic potential (EPSP) large enough to reach the firing threshold in the inhibitory interneurons, and thus no SICI would occur. In contrast, if two CS are applied with short interstimulus intervals, the EPSP evoked by the second CS could summate with the EPSP evoked by the first CS so that the threshold for SICI was reached.

The results of experiment 3 were consistent with this idea of temporal facilitation since they showed that two CS_PA_ stimuli (at 2 and 3 ms) produce an effect larger than the predicted sum of each stimulus alone. However, temporal facilitation was not evident when CS_***AP3***_ was paired with CS_PA2_. Importantly, the amount of SICI produced by CS_***AP3***_ alone was the same as when CS_PA3_ was used, and thus we might have expected to see the same amount of temporal summation with CS_PA2_. In fact, inhibition was only equal to the expected sum of the effect of each CS alone. This is consistent with the idea that CS_PA_ and CS_***AP***_ have an additive effect such as would be expected from activating two separate inhibitory circuits. This does not necessarily mean that they activate two different sets of inhibitory interneurons. It could be, for example, that excitatory inputs activated by CS_PA_ and CS_***AP***_ activate different fractions of the same inhibitory population, or that their inputs target different, non-interacting parts of the dendritic tree of a single neuron. Whatever the mechanistic details, the result suggests that inhibition by CS_***AP***_ at 3 ms is not a time-delayed version of CS_PA2_.

Note that our CS intensities were above the threshold for producing some SICI and therefore slightly higher than in the original experiments of Bestmann et al. [19]. However, any discharge of inhibitory neurones by the first stimulus would have made them refractory to the second stimulus, and if anything, this would have underestimated any temporal facilitation occurring within the subliminal fringe of non-activated neurones.

### 4.5 Interaction of CBI and SICI

The interaction of CBI and SICI_CSPA2_ was first examined by Daskalakis et al. [20]. As in the present study, they found that combined stimulation did not produce more inhibition than either CS alone. However, in their experiments, they described less inhibition, or even facilitation, when both CS were applied. One possible reason that we failed to observe a significant reduction in inhibition is that we used SICI at 3 ms, whereas they used 2 ms. In addition, we express the amount of inhibition as a percent of the response to the TS alone. However, Daskalakis used a test amplitude of 0.5 mV to assess SICI alone and CBI alone, but in order to assess the combination of CBI + SICI, they increased the intensity of the test stimulus such that it produced a 0.5 mV MEP in the presence of CBI.

Although we did not perform a condition with this change in test intensity, we can still analyse the data similarly to Daskalakis et al. by expressing the amplitude of the CBI-SICI conditioned response as a percent of the amplitude of the conditioned response to CBI alone. Fig. 7A shows that this type of analysis appears to reduce the amount of SICI_PA_ when measured in the presence of CBI (see supplementary material 6).

The crucial finding, however, was that the effect of combining CBI with CS_***AP3***_ was completely different: there was more inhibition from combined stimulation than to each stimulus alone. In fact, the expected sum of CBI and CS_***AP3***_ was equal to the combined effect of each stimulus alone. As above, this is consistent with the idea that the two independent inhibitory pathways converge on corticospinal output. In contrast, the pathways responsible for CBI and SICI_CSPA3_ interact with each other, as suggested by Daskalakis et al. [20]. This reinforces the conclusion that CS_***AP3***_ and CS_PA3_ produce SICI via different pathways.

### 4.6 Interaction of SAI and SICI

These results and their interpretation are very similar to those for CBI. The combination of SAI and CS_PA3_ produced a similar amount of inhibition to each stimulus given alone, whereas SAI-CS_***AP3***_ produced more inhibition than SICI_***CSAP3***_. As above, this suggests that inhibition produced by SAI and SICI_***CSAP3***_ co-exist as two separate effects on corticospinal excitability. Indeed, inhibition produced by combined stimulation was approximately the same as the expected sum of each conditioning stimulus alone. Interaction of SAI and conventional (CS_PA_) SICI has been investigated previously by Alle et al. and Udupa et al. [21,23]. They found that the SICI became facilitation when SAI presented but the effect of combined stimulation varied with the intensity of the CS and the interstimulus interval used for SICI. Stimuli like those used here produced similar results (Fig. 7B). However, no combination of stimulus parameters produced more inhibition than either stimulus alone, as we observed with SAI-CS_***AP3***_. We conclude that the pathways responsible for SAI and SICI_CSPA3_ interact with each other, whereas those responsible for SAI and SICI_***CSAP3***_ are likely to be independent.

### 4.7 The role of different inhibitory circuits in behaviour

The results of experiment 6 show for the first time that distinct SICI circuits are differentially modulated throughout the time course of movement preparation. The implication is, again, compatible with the hypothesis that CS_PA_ and CS_***AP***_ probe different inhibitory circuits and that these circuits are modulated at specific times during the course of movement. The effects do not differ across the effector probed and are state-dependent, as no differences were found across rest or tonic activation. While speculative, changes in SICI probed with CS_***AP***_ may reflect the influence of premotor area [7,38] which are modulated earlier in the reaction period than the circuits probed by CS_PA_, which may reflect direct involvement of M1 [4,6].

What is activated by different directions of CS?

The results are consistent with the notion that opposite directions of CS activate two different populations of inhibitory neurons, but they give no information about where and what these neurons are. Nevertheless, they do give some clues.

1) The threshold for producing inhibition is the same in each direction when ISI is 3 ms or longer.
2) Inhibition starts at shorter ISIs with CS_PA_ compared with CS_***AP***_**.**

**Fig. 9:**
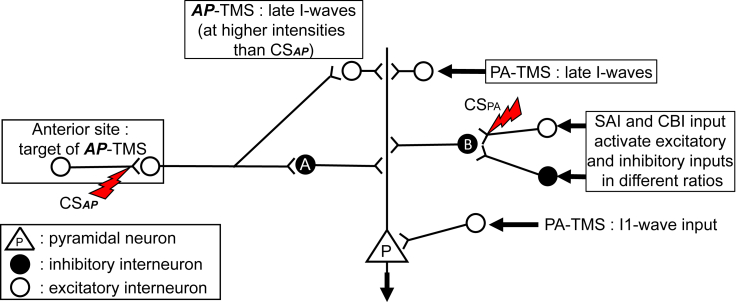
Possible neural circuit to account for the interactions between CS_***AP***_ and CS_PA_ and other forms of cortical inhibition. Neuron P is a large pyramidal cell in the motor cortex with an axon projecting to spinal cord. It receives excitatory (open circles) and inhibitory (filled circles) synaptic inputs, representing I-wave inputs and SICI, respectively. Following recent modelling (Aberra et al. [6]), we assume the lowest threshold sites of activation are presynaptic terminals. The lightning bolts represent the sites activated by subthreshold CS_PA_ and CS_***AP***_. The former activates excitatory inputs to a GABAa-ergic inhibitory interneuron (B) that causes SICI_CSPA_. The latter activates synapses in a more rostral location (e.g., premotor cortex) that activate a different set of GABAa-ergic inhibitory interneurons (A) that produce SICI_***CSAP***_. Neuron B also receives excitatory and inhibitory inputs from SAI and CBI that are responsible for the interaction of these forms of inhibition with SICI_CSPA_. However, they have no direct effect on neuron A which mediates SICI_***CSAP***_. Higher intensities of TMS activate excitatory inputs: PA-TMS activates I1-wave input to the proximal portion of neuron P at a site where it is relatively unaffected by dendritic SICI. Later I-wave inputs, activated by PA-TMS or ***AP***-TMS, are shown as targeting dendritic sites near to the SICI inputs.

In addition, since there are no long-range inhibitory connections in the cortex, the neurons that inhibit the MEP are likely to be near to the corticospinal neurones that conduct the final motor output to the cord. Possibilities include neurons that monosynaptically inhibit corticospinal neurons (i.e. direct inhibition) or neurons that inhibit excitatory inputs to corticospinal neurons (i.e. dis-facilitation) (Fig. 9). Realistic neuronal modelling [6] favours the former, suggesting that inhibition produced by CS_PA_ is caused by stimulation of synaptic terminals of basket cells that monosynaptically inhibit corticospinal neurons and/or activation of excitatory synapses onto the basket neurons (as in Fig. 9).

The same models suggest that ***AP***-TMS (single-pulse TMS with suprathreshold intensity in ***AP*** direction) shifts the site of neural activation anteriorly compared with PA-TMS (single-pulse TMS with suprathreshold intensity in PA direction). If CS_***AP***_ activates at a more anterior site, it may discharge a cortico-cortical neuron that excites inhibitory neurons in the posterior location near to the corticospinal neurones. Because these inhibitory neurones interact with CBI and SAI in a different way to those activated by CS_PA_, they may represent a different population (signified in Fig. 9 as neurones A and B). Conduction delay and an extra synapse might account for the delay (3 ms) in the onset of inhibition. But why would the threshold for evoking SICI from this anterior site be the same as for CS_PA_, particularly since the threshold for evoking ***AP***-MEPs is higher than for PA-MEPs? One possibility relates to the fact that ***AP***-TMS recruits late I-waves, which could have a high threshold and require a greater amount of excitatory input to discharge than small, inhibitory interneurons responsible for SICI. Thus the ***AP*** stimulus intensity required to produce sufficient excitatory input to generate an MEP would be greater than that required to produce inhibition. Another factor that might raise the threshold for ***AP***-MEPs is that AP stimulation does not recruit I1 waves [38]; MEPs only occur when the stimulus intensity is sufficient to evoke late I-waves. In contrast, PA stimulation recruits I1 waves at a lower intensity than I3 waves. These I1 waves can evoke an MEP during a background voluntary contraction. The result is that AMT_PA_ is lower than AMT_***AP***_, whereas the thresholds may be similar for SICI.

However, there are alternative explanations. It could be that the anterior site targeted by CS_***AP***_ provides background excitatory input to the posterior site (e.g. AP-TMS might target premotor cortex while PA-TMS might target M1 [6,7,38]). In this case, SICI_***CSAP***_ could evoke local inhibition in the premotor cortex and remove ongoing facilitation from M1.

### 4.8 Limitations

The experiments used a limited range of stimulus intensities and interstimulus intervals. Thus, our conclusions are also limited to the parameters we have investigated. It is possible that, with other parameters, the effects of CS_***AP3***_ and CS_PA3_ may be more similar, and it will be important to perform more studies in future in order to know if it is possible to generalize the conclusions. For example, by analogy with the latency difference between MEPs evoked by ***AP*** and PA test pulses, it is possible that inhibition using CS_***AP***_ is a delayed version of CS_PA_. Thus, it could be argued that we should have compared CS_***AP3***_ with earlier timings of CS_PA_ rather than CS_PA3_. Udupa et al. [23] found that there were subtle differences in the way CS_PA2_ and CS_PA3_ interacted with SAI, but such effects would not be sufficient to explain the very different interactions we saw here.

A second limitation of the present study was the potential contamination of SICI at ISI = 3 ms by short-interval intracortical facilitation (SICF). Using two pulses of the same direction, the second peak of SICF occurs at around 2.8 ms [39], and a similar timing was noted for pulses of opposite direction [40] (although Delvendahl and coworkers [40] used biphasic pulses for this part of their experiment). Nevertheless, we think any interaction would have been minimal since, when SICF is present, it usually shows up as a reduction in inhibition at higher intensities of CS [39]. When we used an ***AP*** conditioning stimulus to suppress a PA test response (Fig. 2B), reduced inhibition only occurs at around 80% MSO (in the ***AP*** direction), which again is much higher than the range of CS intensities we used in the experiments.

The experiments were also limited technically by the fact that we could not randomize the direction of the CS from trial to trial and had to use two separate, overlapping coils, rather than a single one. Achieving these would probably have reduced the variability of the results, but we think it unlikely to change the main conclusions. Finally, we note that the differences between PA and ***AP*** conditioning stimuli differ between individuals, which probably indicates that it is not possible with a TMS pulse to isolate completely one set of inhibitory neurones from another.

## 5 Conclusion

We show that different directions of TMS-induced currents in the brain are capable of recruiting two independent sets of inhibitory inputs over a range of ISIs used to probe SICI. Fig. 9 illustrates a possible mechanism consistent with the results of this and other studies [6,[14], [15], [16],^20^,^21^,^23^,^38^]. It will be interesting in future studies to test whether these two inhibitory pathways are preferentially active in different types of movement and are affected differently in neurological disorders.

## CRediT authorship contribution statement

**Po-Yu Fong:** Conceptualization, Methodology, Formal analysis, Investigation, Writing – original draft, Writing – review & editing, Visualization. **Danny Spampinato:** Conceptualization, Methodology, Formal analysis, Investigation, Writing – original draft, Writing – review & editing, Visualization. **Lorenzo Rocchi:** Conceptualization, Methodology, Writing – review & editing. **Ricci Hannah:** Conceptualization, Methodology, Writing – review & editing. **Yinghui Teng:** Writing – original draft, Investigation. **Alessandro Di Santo:** Investigation. **Mohamed Shoura:** Investigation. **Kailash Bhatia:** Conceptualization, Resources, Supervision. **John C. Rothwell:** Conceptualization, Methodology, Resources, Writing – review & editing, Supervision, Funding acquisition.

## Declaration of competing interest

The authors declare that they have no known competing financial interests or personal relationships that could have appeared to influence the work reported in this paper.

## Appendix A Supplementary data

Supplementaty material 1 Supplementaty material 1

Supplementaty material 2 Supplementaty material 2

Supplementaty material 3 Supplementaty material 3

Supplementaty material 4 Supplementaty material 4

Supplementaty material 5 Supplementaty material 5

Supplementaty material 6 Supplementaty material 6

Supplementaty material 7 Supplementaty material 7

Supplementary material 8- Legend of supplementary figures Supplementary material 8- Legend of supplementary figures
