# Supplementary material for: Two forms of short-interval intracortical inhibition in human motor cortex
Source: Brain Stimul. 2021 Sep-Oct;14(5):1340–52. doi: 10.1016/j.brs.2021.08.022 (PMC8460995; doi:10.1016/j.brs.2021.08.022)
Supplement: Supplementaty material 1 [file mmc1.docx]

**Supplementary Material**

**Supplementary data I**

**Measurement of the waveform from overlapped coils**

To check that overlapping the coils did not affect the stimulus waveform, we used a pickup coil (Search Coil, radius 6.18mm, Magstim, UK) to record outputs from each coil alone and then when overlapped. Waveforms at intensity of 50% maximum stimulator output (MSO) were recorded with a digital oscilloscope (Owon, 100MHz, Fujian Lilliput Optoelectronics Technology Co., Ltd., China). A 3cm distance between the centre of the bottom of the oval coil to the edge of pickup coil produced the best resolution on the screen of oscilloscope. When testing D50 coil alone, the distance between pickup coil and the centre of the bottom of D50 coil was about 4.5cm (Fig. S1A).

Then TMS-induced current waveforms were processed with the software Oscilloscope (Version 2.2.2, Owon, Fujian Lilliput Optoelectronics Technology Co., Ltd, China) to calculate values in amplitude (mV) and time (µs) and to plot the waveform as well (Fig. S1B-E).
