# Supplementary material for: Two forms of short-interval intracortical inhibition in human motor cortex
Source: Brain Stimul. 2021 Sep-Oct;14(5):1340–52. doi: 10.1016/j.brs.2021.08.022 (PMC8460995; doi:10.1016/j.brs.2021.08.022)
Supplement: Supplementaty material 2 [file mmc2.docx]

**Supplementary data II**

**Combining results from Exp 1 and Exp 2 to explore the effect of CSPA and CSAP at ISI of 2-3ms**

Experiments 1 and 2 both explored the effects of CS_PA_ and CS***_AP_*** at ISI of 2 and 3ms. Since 6 of the participants in experiment 1 did not participate in experiment 2, they could be added to the 15 in experiment 2 to increase strength of conclusions at the two different ISIs. A two-way RM-ANOVA with “Orientation” (CS_PA_ and CS***_AP_***) and “ISI” (2ms and 3ms) as main factors in these 21 individuals showed a significant “Orientation x ISI” interaction (F_1,20_=15.235, p=0.001, Fig. S2). This was due to the fact that CS***_AP2_*** produced much less SICI than CS***_AP3_*** (p=0.023), whereas there was no obvious difference in SICI evoked by CS_PA2_ and CS_PA3_ (p=0.053). A post hoc comparison showed that CS***_AP2_*** induced much less SICI than CS_PA2_ (p=0.001). These results confirm that although CS***_AP_*** and CS_PA_ produce the same amount of inhibition (at the same absolute intensity of CS) at ISI = 3 ms, CS***_AP_*** is much less effective at ISI = 2 ms.
