# Supplementary material for: Two forms of short-interval intracortical inhibition in human motor cortex
Source: Brain Stimul. 2021 Sep-Oct;14(5):1340–52. doi: 10.1016/j.brs.2021.08.022 (PMC8460995; doi:10.1016/j.brs.2021.08.022)
Supplement: Supplementaty material 3 [file mmc3.docx]

**Supplementary data III**

**Effects of state of contraction on the amplitude of the test MEP in experiment 6**.

Rest vs tonic contraction: In the FDI muscle, the amplitude of test MEPs in blocks of trials testing SICI_CSPA3_ and SICI***_CSAP3_*** were all identical for all contraction states. A two-way RM-ANOVA with “Orientation” (SICI_CSPA3_ and SICI***_CSAP3_***) and “Brain state” (rest and tonic muscle contraction) revealed no “Orientation x Brain state” interaction (F_1,12_=0.070, p=0.796), and no main effect of “Brain state” (F_1,12_=0.235, p=0.636), or “Orientation” (F_1,12_=0.586, p=0.459) (Fig. S3A). The absence of an effect of “Brain state” is expected since the test intensity was adjusted to produce an approximate 1 mV MEP in FDI both at rest and during contraction. However, the size of test MEP of ADM was larger during tonic contraction than at rest. A two-way RM-ANOVA with “Orientation” (SICI_CSPA3_ and SICI***_CSAP3_***) and “Brain state” (rest and tonic muscle contraction) showed a main effect of “Brain state” (F_1,12_=29.254, p<0.001), but there was no significant “Orientation x Brain state” interaction (F_1,12_=0.367, p=0.556) and no main effect of “Orientation” (F_1,12_=1.303, p=0.276) (Fig. S3B).

MEPs during SRTT: As expected, the amplitude of test MEP in FDI increased when evoked during the reaction period (at RT_35%_ and RT_70%_). A two-way RM-ANOVA with “Orientation” (SICI_CSPA3_ and SICI***_CSAP3_***) and “Time” (on the cue, 35%RT, 70% RT) as main factors was applied to investigate change of MEP amplitude in each muscle. In the session of FDI, there was a significant effect of “Time” in the two-way RM-ANOVA (F_1.058,12.694_=18.164, p=0.001) but no “Orientation x Time” interaction (F_1.027,12.323_=0.062, p=0.813) and no main effect of “Orientation” (F_1,12_=0.062, p=0.807). Post hoc analysis revealed that the test MEP was smallest at the time of the auditory cue (p=0.022 and p=0.002, at RT_35%_ and RT_70%_ respectively). In addition, the MEP was larger at RT_70%_ than at RT_35%_ (p=0.007) (Fig. S3C). Finally, the test MEP in ADM was the same at all the three time points. A two-way RM-ANOVA revealed no “Orientation x Time” interaction (F_2,24_=0.561, p=0.578), and no main effects of “Orientation” (F_2,24_=0.275, p=0.123) or time (F_2,24_=0.444, p=0.646)) (Fig. S3D).
