# Supplementary material for: Two forms of short-interval intracortical inhibition in human motor cortex
Source: Brain Stimul. 2021 Sep-Oct;14(5):1340–52. doi: 10.1016/j.brs.2021.08.022 (PMC8460995; doi:10.1016/j.brs.2021.08.022)
Supplement: Supplementaty material 4 [file mmc4.docx]

**The reason about unequal numbers of participants among experiments**

In experiment 1, a previous study (Hanajima et al. 2008) had shown that SICI***_CSAP-TSAP_*** at 2ms was significantly weaker than 3ms, so we decided to test only a small number of participant (n=11) to confirm this phenomenon. To replicate this phenomenon and explore other ISIs as well, we tested more participants (n=15) in experiment 2. The result of experiment 2 did replicate the same result in experiment 1 and also indicated that SICI***_CSAP1_*** was weaker than SICI_CSPA1_. Based on previous studies using triple pulse TMS (SICI, CBI and SAI) and integrating SRTT [1], we aimed to recruit 16 subjects but the COVID-19 pandemic forced our lab to close. Since the results were very clear we decided to proceed with publication with the following final numbers: experiment 3 (n=13), 4 (n=16), 5 (n=14), and 6 (n=13).

[1] Hannah R, Cavanagh SE, Tremblay S, Simeoni S, Rothwell JC. Selective Suppression of Local Interneuron Circuits in Human Motor Cortex Contributes to Movement Preparation. J Neurosci 2018;38(5):1264-76.
