# Supplementary material for: Two forms of short-interval intracortical inhibition in human motor cortex
Source: Brain Stimul. 2021 Sep-Oct;14(5):1340–52. doi: 10.1016/j.brs.2021.08.022 (PMC8460995; doi:10.1016/j.brs.2021.08.022)
Supplement: Supplementaty material 5 [file mmc5.docx]

**The overlap of participants between each experiment**

We listed the overlap of participants below.

| Experiment and participants | Experiment and participants | Overlap |
| --- | --- | --- |
| Experiment 1 (n=11) | Experiment 2 (n=15) | 5 subjects |
| Experiment 1 (n=11) | Experiment 3 (n=13) | 4 subjects |
| Experiment 1 (n=11) | Experiment 4 (n=16) | 5 subjects |
| Experiment 1 (n=11) | Experiment 5 (n=14) | 6 subjects |
| Experiment 1 (n=11) | Experiment 6 (n=13) | 2 subjects |
| Experiment 2 (n=15) | Experiment 3 (n=13) | 13 subjects |
| Experiment 2 (n=15) | Experiment 4 (n=16) | 11 subjects |
| Experiment 2 (n=15) | Experiment 5 (n=14) | 6 subjects |
| Experiment 2 (n=15) | Experiment 6 (n=13) | 6 subjects |
| Experiment 3 (n=13) | Experiment 4 (n=16) | 10 subjects |
| Experiment 3 (n=13) | Experiment 5 (n=14) | 5 subjects |
| Experiment 3 (n=13) | Experiment 6 (n=13) | 7 subjects |
| Experiment 4 (n=16) | Experiment 5 (n=14) | 6 subjects |
| Experiment 4 (n=16) | Experiment 6 (n=13) | 3 subjects |
| Experiment 5 (n=14) | Experiment 6 (n=13) | 2 subjects |
