# Supplementary material for: Two forms of short-interval intracortical inhibition in human motor cortex
Source: Brain Stimul. 2021 Sep-Oct;14(5):1340–52. doi: 10.1016/j.brs.2021.08.022 (PMC8460995; doi:10.1016/j.brs.2021.08.022)
Supplement: Supplementaty material 6 [file mmc6.docx]

**Interaction between SICI_CSPA3_ and other inhibitory circuits (CBI, SAI) in triple pulses stimulation**

Here we examined the change of SICI_CSPA3_ when CBI or SAI presented. In previous report by Daskalakis et al., they investigated how CBI affects SICI_CSPA2_ in triple pulse stimulation. In their condition A of figure 3, the intensity for the test stimulation of SICI_CSPA2_ could reach 0.5mV of amplitude of MEP. The ratio of SICI_CSPA2_ was conditioned MEP/test MEP_0.5mV_. In their condition B of figure 3, the intensity for the test stimulation of SICI_CSPA2_ (not giving CBI together) could produce 0.5mV in CBI protocol. The ratio of SICI_CSPA2_ was conditioned MEP/test MEP_CCS0.5mV_. In their condition C of figure 3, the intensity for test stimulation in triple pulse stimulation (giving CBI together) was the same as the second condition. The ratio of SICI change was conditioned MEP of CBI-CS_PA2_ /conditioned MEP of CBI. The test MEP for both CBI-CS_PA2_ and CBI were test MEP_CCS0.5mV_. There results showed SICI_CSPA2_ was suppressed significantly when CBI presented by comparing condition C and condition B. And similar trend was also observed by comparing condition C and condition A.

In contrast, because our study set the intensity for test stimulation was to reach 1mV across all conditions, here we only have two conditions to compare. One is the condition of SICI_CSPA3_ and ratio was conditioned MEP/test MEP_1mV_. The other one is triple pulse stimulation and the change of SICI ratio (1.36 ± 0.59) was conditioned MEP of CBI-CS_PA3_ / conditioned MEP of CBI. The test MEP for both CBI-CS_PA3_ and CBI were test MEP_1mV_. Both ratios fit normality by Shapiro-Wilk test. The comparison between the two conditions is similar to the comparison between condition B and condition C in figure 3 in the paper by Daskalakis et al. A paired t test revealed CBI suppressed SICI_CSPA3_ significantly ( t=-4.128, df=15, p=0.001), and the result is consistent to their result.

When we applied the same way to examine how SAI affect SICI_CSPA3_ in triple pulse stimulation. Again, we have two conditions to compare here. One is the condition of SICI_CSPA3_ and ratio was conditioned MEP/test MEP_1mV_. The other one is triple pulse stimulation and the change of SICI ratio (2.13 ± 0.29) was conditioned MEP of SAI-CS_PA3_ / conditioned MEP of SAI. The test MEP for both SAI-CS_PA3_ and SAI were test MEP_1mV_. Both ratios fit normality by Shapiro-Wilk test. A paired t test revealed SICI_CSPA3_ became facilitated significantly when SAI presented (t=-4.441, df=13, p=0.001) like the results of the experiments by Udupa et al.
