# Supplementary material for: Two forms of short-interval intracortical inhibition in human motor cortex
Source: Brain Stimul. 2021 Sep-Oct;14(5):1340–52. doi: 10.1016/j.brs.2021.08.022 (PMC8460995; doi:10.1016/j.brs.2021.08.022)
Supplement: Supplementaty material 7 [file mmc7.docx]

**Pilot study: SICI*_CSAP3_* using different intensites of conditioning stimulation to interact with SAI**

We conducted this experiment to determine whether SICI***_CSAP_*** evoked with a higher intensity of CS would behave differently. During the pandemic, it was not easy to find and test healthy participants. In the end, eight healthy volunteers (22.4 ± 2.6 years old, all female) were recruited. We compared SICI and its interaction with SAI using CS***_AP3_*** at 90% AMT***_AP_*** (CS***_AP3H_***: relative threshold) and CS***_AP3_*** at 90% AMT_PA_ (CS***_AP3L_***: absolute intensity). Conditions included SAI lone (ISI 22ms), SICI***_CSAP3H_*** alone(with 90% AMT***_AP_***), SICI***_CSAP3L_*** alone (with 90% AMT_PA_), and SAI-SICI***_CSAP3H_***, SAI-SICI***_CSAP3L_***. The design of this experiment was as in Experiment 5. SAI was tested in an independent block. Another test block included 3 conditions: Test stimulation, SICI***_CSAP3H_*** and SAI-SICI***_CSAP3H_***. The other test block included 3 conditions: Test stimulation, SICI***_CSAP3L_*** and SAI-SICI***_CSAP3L_***. Each condition had 20 trials. The intensity for test stimulation was adjusted to produce a 1mV in peak to peak MEP.

Regarding statistics, a two-way RM-ANOVA with Intensity (level: CS***_AP3H_*** and CS***_AP3L_***) and Condition (level: SICI and triple pulse) as main factors was used. Mauchly’s test was used to check sphericity in this two-way RM-ANOVA, and Greenhouse-Geisser correction was used for non-sphericity conditions. Post hoc paired comparisons with Bonferroni multiple correction was calculated.

The results are illustrated in the Fig.S4. As expected, a two-way RM-ANOVA revealed a significant interaction (F_1,7_=9.182, p=0.019). Post hoc paired comparisons showed that CS***_AP3H_*** evokes more powerful SICI than CS***_AP3L_*** (p = 0.048). In addition, the triple pulse experiments show that the interaction of SAI and SICI produces more inhibition than SICI alone using CS***_AP3L_*** by another post hoc paired comparison (p = 0.030), but that this is not the case using CS***_AP3H_*** (Fig.S4).
