# Supplementary material for: Two forms of short-interval intracortical inhibition in human motor cortex
Source: Brain Stimul. 2021 Sep-Oct;14(5):1340–52. doi: 10.1016/j.brs.2021.08.022 (PMC8460995; doi:10.1016/j.brs.2021.08.022)
Supplement: Supplementary material 8- Legend of supplementary figures [file mmc8.docx]

**Fig. S1:** Waveform measurement.

The setup of waveform recording with pick-up coil and oscilloscope (A). The waveform of oval coil firing when non-overlapped (B) and overlapped (C). The waveform of the D50 coil when non-overlapped (D) and overlapped (E). There were no reversed monophasic waveforms or transformed waveforms in these recordings, which means there was no induced current in another coil. The amplitudes of waveforms in each coil were slightly different because the distance between the pick-up coil and the two coils could be slightly moved away from the original position after we bind the two coils together.

**Fig. S2:** CS***_AP_*** vs CS_PA_ at ISI 2-3ms with 21 subjects

There was still a strong significant difference by interaction with “Orientation” and “ISI” when reinforced the number of subjects up to 21 subjects. Asterisk indicated significant interaction by two-way RM-ANOVA (p<0.05). Double asterisk means significant difference by post hoc study (p<0.05).

**Fig. S3:** Test MEPs in behaviour task

Left column (A,C) plotted the test MEP of FDI SICI and right column (B,D) illustrated the test MEP of ADM SICI. The amplitude of test MEPs within SICI_CSPA3_ and SICI***_CSAP3_*** were not different at rest or during tonic movement by either FDI (A) or ADM (B), although there was a significant increasing in size of MEP from rest to tonic movement by ADM (*). In session of SRTT, the amplitude of test MEP between SICI_CSPA3_ and SICI***_CSAP3_*** within the same condition were still not statistical different. Elevated FDI test MEP just before movement was noted (*) whereas this did not happen at ADM test MEP. This is because we only use FDI to do SRTT. Asterisk indicated significance (p<0.05) in factor of brain state within a two-way RM-ANOVA in panel B and factor of time within a two-way RM-ANOVA in panel C.

**Fig. S4: Different intensity of conditioning stimulus for SICI*_CSAP3_***

The left panel shows the amount of inhibition produced by SAI alone (SAI; ISI = 22 ms). The middle panel represents SICI obtained with CS***_AP3L_***, either alone or combined with ulnar nerve stimulation (SAI-CS***_AP3L_***). The right panel plots SICI induced by CS***_AP3H_*** alone and combined with ulnar nerve stimulation (SAI-CS***_AP3H_***). SICI***_CSAP3H_*** produced stronger inhibition than SICI***_CSAP3L_***. However, the triple pulse combining SAI and CS***_AP3L_*** behaved differently from that of SAI-CS***_AP3H_***. Double asterisks indicate p<0.05 in post hoc pairwise comparison. CS1 indicates the earlier CS, and CS2 means the later CS.
