# Supplementary figures and images for: Two forms of short-interval intracortical inhibition in human motor cortex
Source: Brain Stimul. 2021 Sep-Oct;14(5):1340–52. doi: 10.1016/j.brs.2021.08.022 (PMC8460995; doi:10.1016/j.brs.2021.08.022)

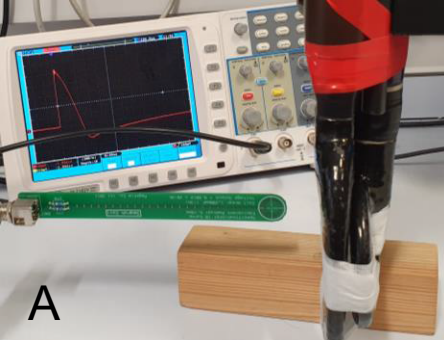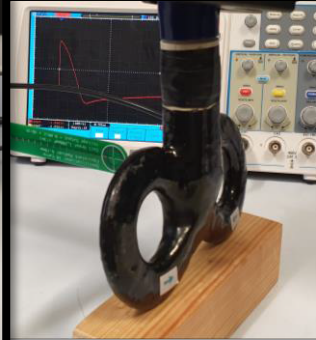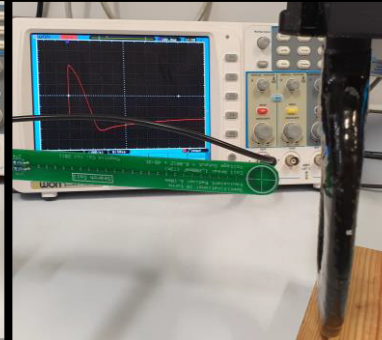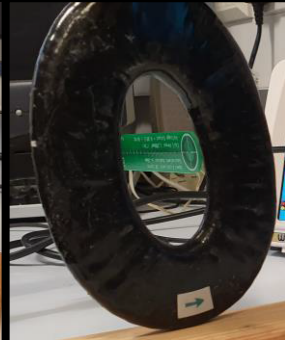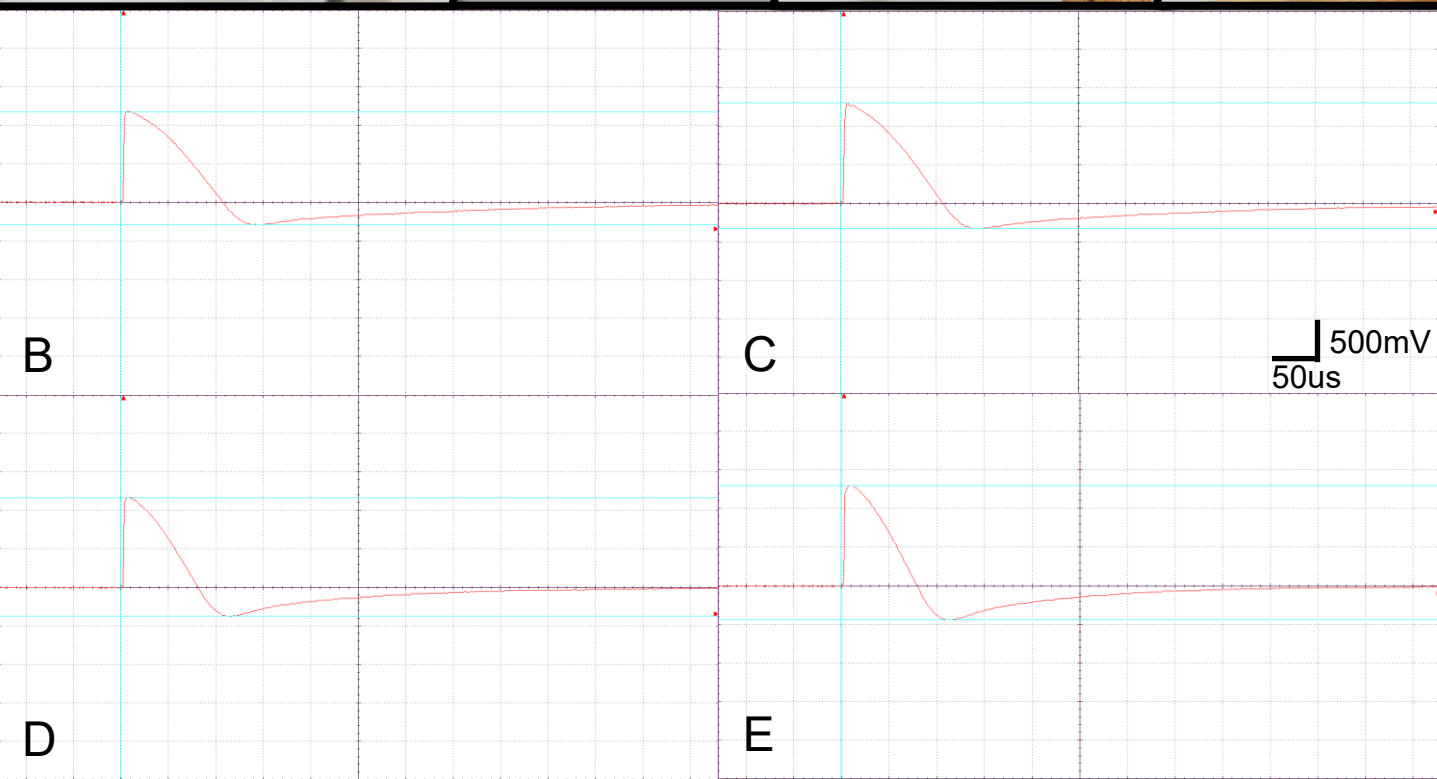

Supplement: Fig. S1 [file mmc9.pdf]

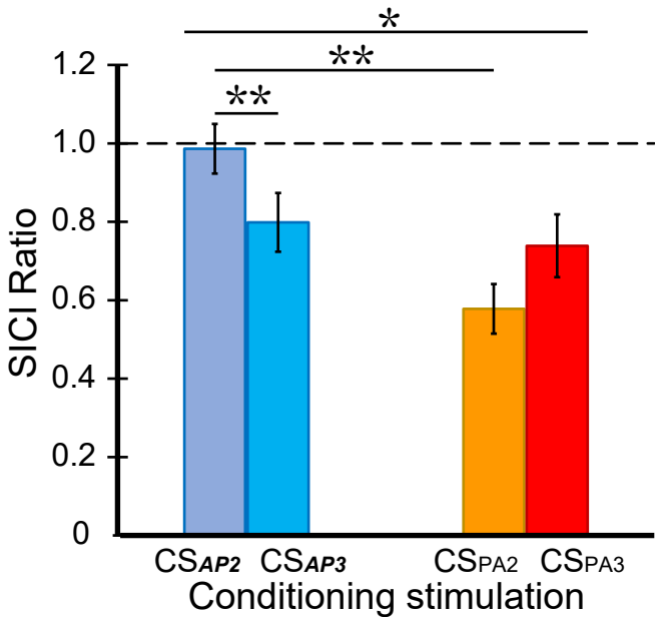

Supplement: Fig. S2 [file mmc10.pdf]

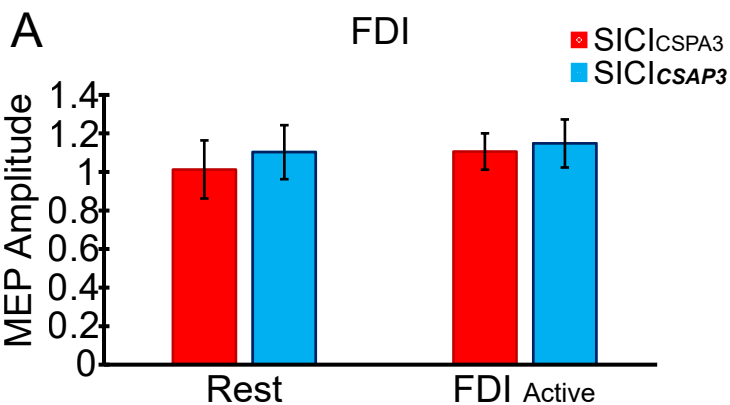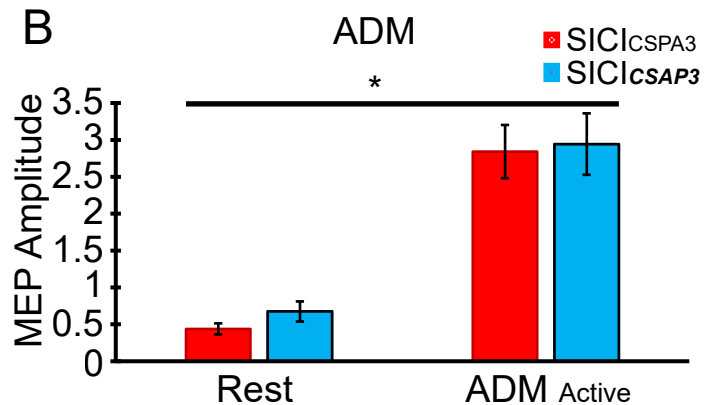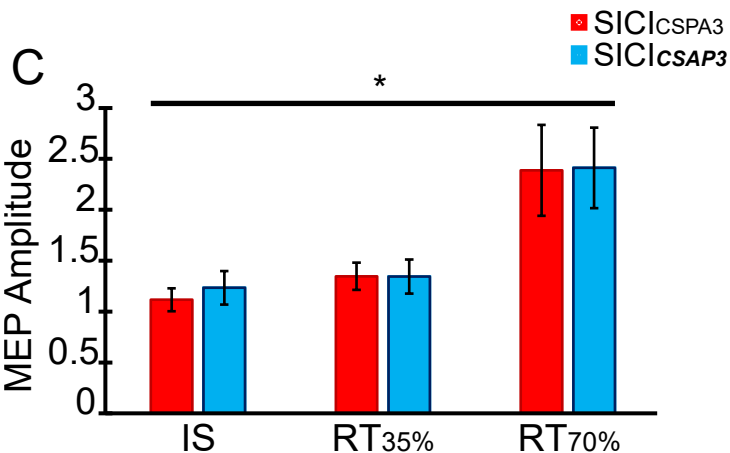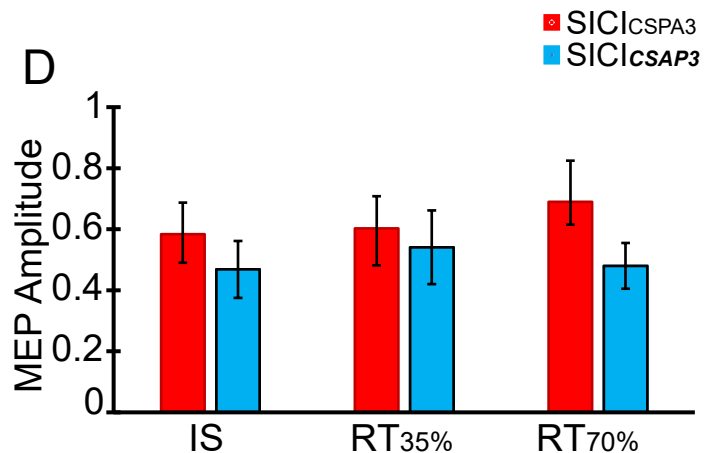

Supplement: Fig. S3 [file mmc11.pdf]

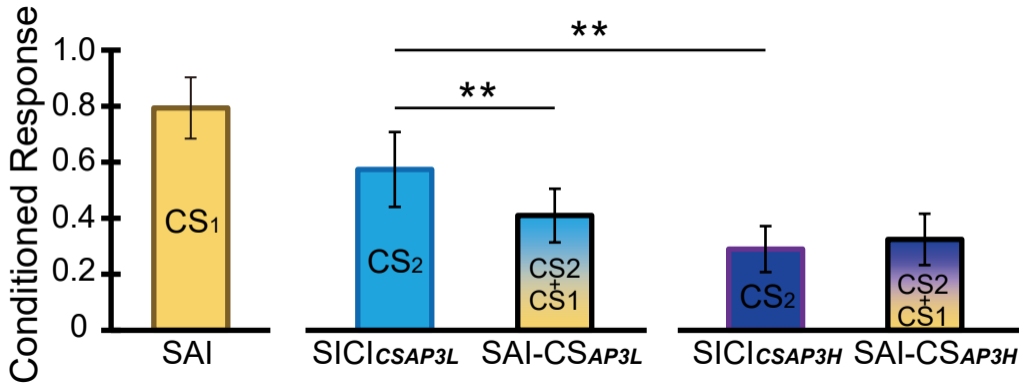

Supplement: Fig. S4 [file mmc12.pdf]
